# Systematic Cell-Based Phenotyping of Missense Alleles Empowers Rare Variant Association Studies: A Case for *LDLR* and Myocardial Infarction

**DOI:** 10.1371/journal.pgen.1004855

**Published:** 2015-02-03

**Authors:** Aenne S. Thormaehlen, Christian Schuberth, Hong-Hee Won, Peter Blattmann, Brigitte Joggerst-Thomalla, Susanne Theiss, Rosanna Asselta, Stefano Duga, Pier Angelica Merlini, Diego Ardissino, Eric S. Lander, Stacey Gabriel, Daniel J. Rader, Gina M. Peloso, Rainer Pepperkok, Sekar Kathiresan, Heiko Runz

**Affiliations:** 1 Institute of Human Genetics, University of Heidelberg, Heidelberg, Germany; 2 Molecular Medicine Partnership Unit (MMPU), University of Heidelberg/ EMBL, Heidelberg, Germany; 3 Center of Human Genetic Research (CHGR), Massachusetts General Hospital, Boston, Massachusetts, United States of America; 4 Broad Institute of MIT and Harvard, Cambridge, Massachusetts, United States of America; 5 Cardiovascular Research Center, Massachusetts General Hospital, Boston, Massachusetts, United States of America; 6 Department of Medicine, Harvard Medical School, Boston, Massachusetts, United States of America; 7 Cell Biology/Biophysics Unit, European Molecular Biological Laboratory, Heidelberg, Germany; 8 Humanitas University, Milan, Italy; 9 Division of Cardiology, Ospedale Niguarda, Milan, Italy; 10 Department of Cardiology, Parma Hospital, Parma, Italy; 11 Perelman School of Medicine, University of Pennsylvania, Philadelphia, Pennsylvania, United States of America; Yale School of Medicine, UNITED STATES

## Abstract

A fundamental challenge to contemporary genetics is to distinguish rare missense alleles that disrupt protein functions from the majority of alleles neutral on protein activities. High-throughput experimental tools to securely discriminate between disruptive and non-disruptive missense alleles are currently missing. Here we establish a scalable cell-based strategy to profile the biological effects and likely disease relevance of rare missense variants *in vitro*. We apply this strategy to systematically characterize missense alleles in the low-density lipoprotein receptor (*LDLR*) gene identified through exome sequencing of 3,235 individuals and exome-chip profiling of 39,186 individuals. Our strategy reliably identifies disruptive missense alleles, and disruptive-allele carriers have higher plasma LDL-cholesterol (LDL-C). Importantly, considering experimental data refined the risk of rare *LDLR* allele carriers from 4.5- to 25.3-fold for high LDL-C, and from 2.1- to 20-fold for early-onset myocardial infarction. Our study generates proof-of-concept that systematic functional variant profiling may empower rare variant-association studies by orders of magnitude.

## Author Summary

Exome sequencing has proven powerful to identify protein-coding variation across the human genome, unravel the basis of monogenic diseases and discover rare alleles that confer risk for complex disease. Nevertheless, two key challenges limit its application to complex phenotypes: first, most alleles identified in a population are extremely rare; and second, most alleles are neutral on protein activities. Consequently, association tests that rely on enumerating rare alleles in cases and controls (termed *rare variant association studies*, RVAS) are typically underpowered, as the many neutral alleles dampen signals that arise from the few alleles that disrupt protein functions. Strategies to securely discriminate disruptive from neutral variants are immature, in particular for missense variants. Here we show that the statistical power of RVAS improves dramatically if variants are stratified according to their *in vitro* ascertained functions. We establish scalable technology to objectively profile the biological effects of exome-identified missense variants in the low-density lipoprotein receptor (*LDLR*) through systematic overexpression and complementation experiments in cells. We demonstrate that carriers of *LDLR* alleles, which our experiments identify as “disruptive-missense”, have higher plasma LDL-C, and that considering *in vitro* data may make it possible to reduce RVAS sample sizes by more than 2-fold.

## Introduction

The rate by which sequencing studies in humans are unraveling genetic variants far outweighs our ability to accurately evaluate which of these variants are of the highest relevance to human health and disease [1]. This interpretative gap is considered a key impediment for the wider use of genetics in clinical medicine [2–4], as it challenges sequencing-based diagnoses [5–7] and risks misguiding medical interventions or reproductive decisions [8]. It further limits the statistical power of sequencing studies in families or populations that aim to identify novel disease genes [9, 10].

The vast majority of rare protein-coding alleles are considered to be neutral, i.e., they have no or little impact on disease liabilities. Importantly, this overabundance of neutral compared with damaging alleles creates a tremendous signal-to-noise problem for rare-variant association studies (RVAS) [10] that rely on the aggregation of all or distinct classes of rare variants at the gene level [11]. RVAS have recently allowed us to identify rare variation in the low-density lipoprotein receptor (*LDLR*) as associated with early-onset myocardial infarction (MI) in the population [12]. Importantly, however, association signals were driven by loss-of-function (LoF) alleles that based on sequence could be unambiguously interpreted as protein-inactivating, including nonsense, splice-site or indel frameshift alleles. Carriers of LoF alleles in *LDLR* showed an 18.1-fold increased MI-risk as opposed to an only 1.7-fold increased risk in carriers of missense alleles. As missense variants by far outnumber LoF variants across human genes [12–14], it has been hypothesized that including disruptive-missense (i.e., missense variants that disrupt protein functions in the range of LoF variants, “missense LoF”), while ignoring neutral alleles should considerably enhance the association signal and reduce the necessary samples sizes needed to demonstrate association, by on average 2.5-fold [10]. However, missense variants are the most difficult class of variants to adequately predict a biological function [15], particularly in genes under selective pressure like *LDLR* where the rate of neutral relative to disruptive-missense alleles is expected to be high [10].

Deleterious variation in *LDLR* is kept at low frequency as heterozygote carriers of mutant alleles show familial hypercholesterolemia (FH), characterized by a 2–3 fold elevation of plasma low-density lipoprotein cholesterol (LDL-C) and premature coronary artery disease [16]. Among Europeans, 4–5% of individuals who suffer from MI before the age of 60 are FH heterozygotes [17]. *LDLR* is also paradigmatic for a dose-response relationship between gene and function as homozygotes are more severely affected than heterozygotes, and mutations that impair, but not completely abolish receptor activity tend to result in more moderately increased LDL-C, later onset MI and better response to therapies [16]. Mutations can impact different activities of the LDLR protein, including its biosynthesis, subcellular trafficking and capacity to bind and internalize LDL [18], yet biochemical tests to characterize FH mutants are low-throughput and not applied routinely in clinical care [19]. Importantly, *LDLR* is one of 56 genes in which the incidental detection of known or novel variants is recommended for subsequent medical clarification [20].

Here we establish an experimental strategy to systematically characterize the biological functions of missense alleles identified through exome analysis of large clinical cohorts. We demonstrate at the case of *LDLR* and MI that a combination of sequencing with systematic variant-profiling *in vitro* markedly improves the statistical power of RVAS.

## Results

### Rare missense alleles deflate association of low-density lipoprotein receptor (*LDLR*) with plasma LDL-C and MI-risk

**Figure 1:**
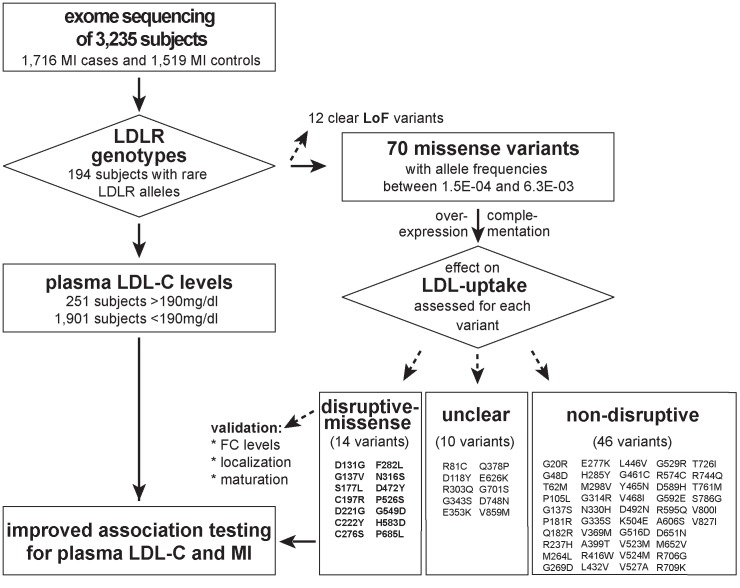
Workflow of this study to determine the functional impact of 70 rare missense variants on LDLR protein activities and improve rare variant association testing for plasma LDL-C and the risk for early-onset MI. Variants were identified through whole-exome sequencing of 3,235 individuals from the Italian Study of Early-onset Myocardial Infarction (ATVB) cohort.

With the aim to identify rare missense alleles in *LDLR* that increase the risk for premature MI, we leveraged the exomes of 1,716 cases with MI prior to age of 46 and 1,519 MI-free controls [12] (see Fig. 1 for workflow of this study). Overall, 194 subjects carried rare *LDLR* alleles that distributed on 12 clear LoF and 70 missense variants (S1 Table, Methods and S1 Spreadsheet). The burden of LoF alleles associated rare variation in *LDLR* with LDL-C and MI-risk at genome-wide significance (p<1×10^-8^) [12]. However, the more abundant missense alleles alone or in combination with LoF variants considerably deflated association signals (e.g., for LDL-C from odds ratio (OR)=34.4 to 3.2 and 4.5, respectively) (Table 1, Tables S2–3). This is consistent with a scenario where the signal of alleles that disrupt LDLR activity—LoF alleles together with missense alleles of a similar impact as LoF alleles (termed “disruptive-missense” alleles)—is swamped by the noise of neutral alleles. *A-priori* information to separate between these two groups is scarce as an overlap of four frequently used computational prediction tools assign equal proportions of *LDLR* missense alleles as damaging (51%) and likely benign (49%), respectively (S1 Table). Moreover, the rate of unique alleles (61%) in the studied at-risk cohort matches that of non-MI reference cohorts (S1 Fig.), which further complicated identification of disruptive-missense alleles from sequence data alone.

**Table 1:** Association of a burden of rare variants in the low-density lipoprotein receptor (*LDLR*) gene with plasma low-density lipoprotein cholesterol (LDL-C) levels and the risk for early-onset myocardial infarction.

**pheno-type**	**variants analyzed**	**variant count**	**allele count**	**allele freq.**	**LDL-C >190mg/dl (n = 251)**	**LDL-C <190mg/dl (n = 1,901)**	**P-value**	**OR**	**95% CI**
**plasma LDL-C**	clear LoF	11	16	0.007	13	3	2×10^-10^	34.4	9.4–189.7
	all missense	55	127	0.059	35	92	4×10^-7^	3.2	2.0–4.9
	all missense + LoF	66	143	0.066	48	95	4×10^-13^	4.5	3.0–6.6
	predicted as damaging	30	48	0.022	22	26	2×10^-9^	6.9	3.7–12.9
	predicted as damaging + LoF	41	64	0.030	35	29	2×10^-17^	10.4	6.1–18.1
	disruptive- missense	13	20	0.009	14	6	1×10^-9^	18.6	6.6–59.6
	disruptive missense + LoF	24	36	0.017	27	9	6×10^-19^	25.3	11.3–61.8
**pheno-type**	**variants analyzed**	**variant count**	**allele count**	**allele freq.**	**MI case (n = 1,716)**	**MI control (n = 1,519)**	**P-value**	**OR**	**95% CI**
**MI**	clear LoF	12	17	0.005	17	0	2×10^-5^	-	3.7-inf.
	all missense	70	177	0.055	119	58	1×10^-4^	1.9	1.4–2.6
	all missense + LoF	82	194	0.060	136	58	8×10^-7^	2.1	1.6–3.0
	predicted as damaging	36	62	0.019	50	12	8×10^-6^	3.8	2.0–7.8
	predicted as damaging + LoF	48	79	0.024	67	12	3×10^-9^	5.1	2.7–10.4
	disruptive- missense	14	29	0.009	27	2	5×10^-6^	12.1	3.0–105.4
	disruptive missense + LoF	26	46	0.014	44	2	2×10^-10^	20.0	5.2–169.9

### Establishment of a microscope-based approach to systematically profile the function of *LDLR* missense alleles

**Figure 2:**
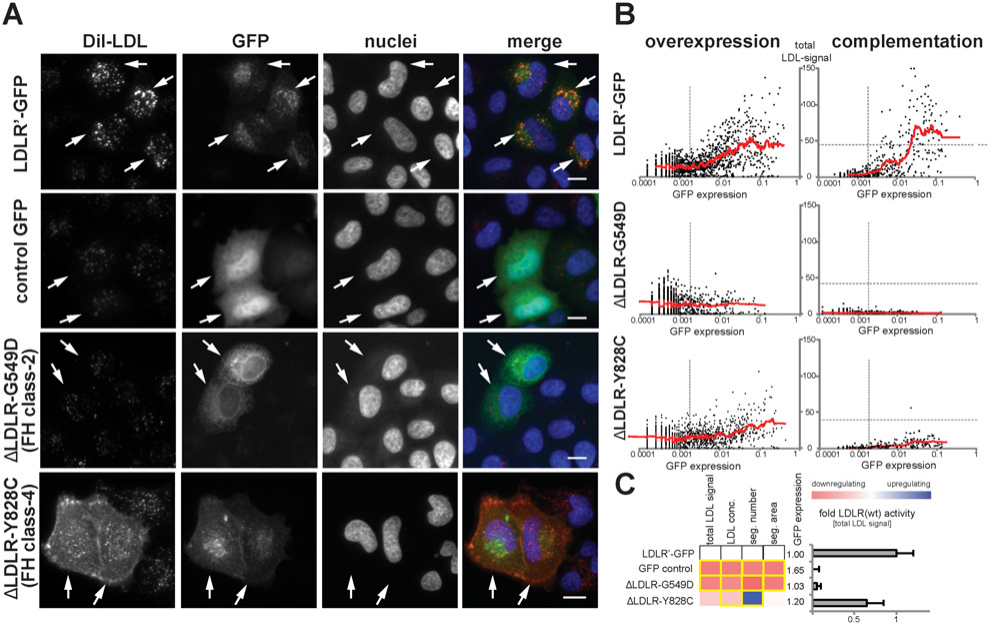
Systematic functional profiling of low-density lipoprotein receptor (*LDLR*) alleles. **(A)** *LDLR* missense variants were functionally characterized by monitoring cellular uptake of fluorescently-labeled LDL (DiI-LDL; *red*) into cells (see Methods). Shown are automatically acquired images of HeLa-Kyoto cells transiently expressing siRNA-resistant full-length human wildtype LDLR linked to EGFP (LDLR’-GFP), empty GFP-control plasmid, or two FH mutants known to inhibit transport (p.G549D; FH class-2) or endocytosis (p.Y828C; FH class-4) of the LDLR protein. Arrows denote GFP-positive cells. Note the localization of FH mutants to different subcellular compartments. Bars = 15μm. **(B)** Graphs depict relative signal intensities of total DiI-LDL in endosome-like subcellular compartments (total LDL signal; y-axis, in arbitrary units) plotted against total cellular GFP expression (x-axis, in arbitrary units) for wildtype LDLR (LDLR’-GFP, upper panel) and indicated FH mutants. Each graph depicts results from a single experimental replica upon either overexpression of the respective cDNA-construct (left graphs) or complementation settings (i.e., siRNA knockdown of endogenous LDLR followed by reconstitution with indicated LDLR-GFP constructs; right graphs). Each dot represents one individual cell. Dashed vertical bars separate cells classified as GFP-negative (left from bar) from cells defined as GFP-positive. Dashed horizontal lines in complementation setting indicate mean total LDL signal in control siRNA-treated cells expressing endogenous LDLR. Cells where total LDL signal fell above this threshold (indicating over-compensation by LDLR’-GFP expression) were not respected for quantifications (see Methods). **(C)** LDLR activity was measured with five phenotypic parameters: total LDL signal in endosome-like compartments, LDL concentration, number (seg. number) and area (seg. area) of subcellular DiI-positive endosome-like structures, and cellular GFP-expression. The heatmap represents means from all experimental replicas per variant under the overexpression setting. Red reflects reduced, blue increased signal relative to wildtype LDLR’-GFP. Phenotypes meeting statistical criteria as described in Methods are framed in orange. Bar graph depicts total LDL signal ±SD normalized to wildtype LDLR’-GFP.

In order to distinguish disruptive from non-disruptive *LDLR* missense alleles, we established a workflow to profile the function of missense alleles in an unbiased, quantitative and high-throughput manner *in vitro*. For this, we applied two complementary experimental strategies: first, an “overexpression” approach where wildtype or mutated LDLR-GFP was transiently expressed in cultured cells; and second a “complementation” approach where the endogenous receptor was silenced with LDLR-siRNA, but receptor activities were reconstituted by co-expressing siRNA-resistant wildtype or mutated LDLR-GFP (S2 Fig. and Methods). Since we assumed that complementation might have the potential to unmask effects that fail to be identified by testing overexpression alone, both approaches were applied in parallel. The efficiency of LDL-uptake into GFP-positive and GFP-negative cells was quantified by multiparametric analyses from images acquired using high-content automated microscopy as described [21, 22] (S3 Fig. and Methods). Expectedly, wildtype LDLR stimulated LDL-uptake, as evidenced by an increased internalization of fluorescent-labeled LDL into endosome-like compartments (Fig. 2A). This effect vanished when LDLR carried the transport–deficient FH-mutation p.G549D [18] that mislocalized the receptor to endoplasmatic reticulum (ER)-like membranes, or the internalization-deficient “JD”-mutant p.Y828C [23] that arrested both, ligand and receptor at the plasma membrane. Multiparametric analysis of the phenotypes obtained from a large number of cells (Fig. 2B,C) demonstrated that our approach could identify and correctly describe functions of previously known *LDLR* missense variants causing FH.

### Functional characterization of rare *LDLR* alleles identified through exome sequencing of 3,235 individuals uncovers disruptive-missense variants

**Figure 3:**
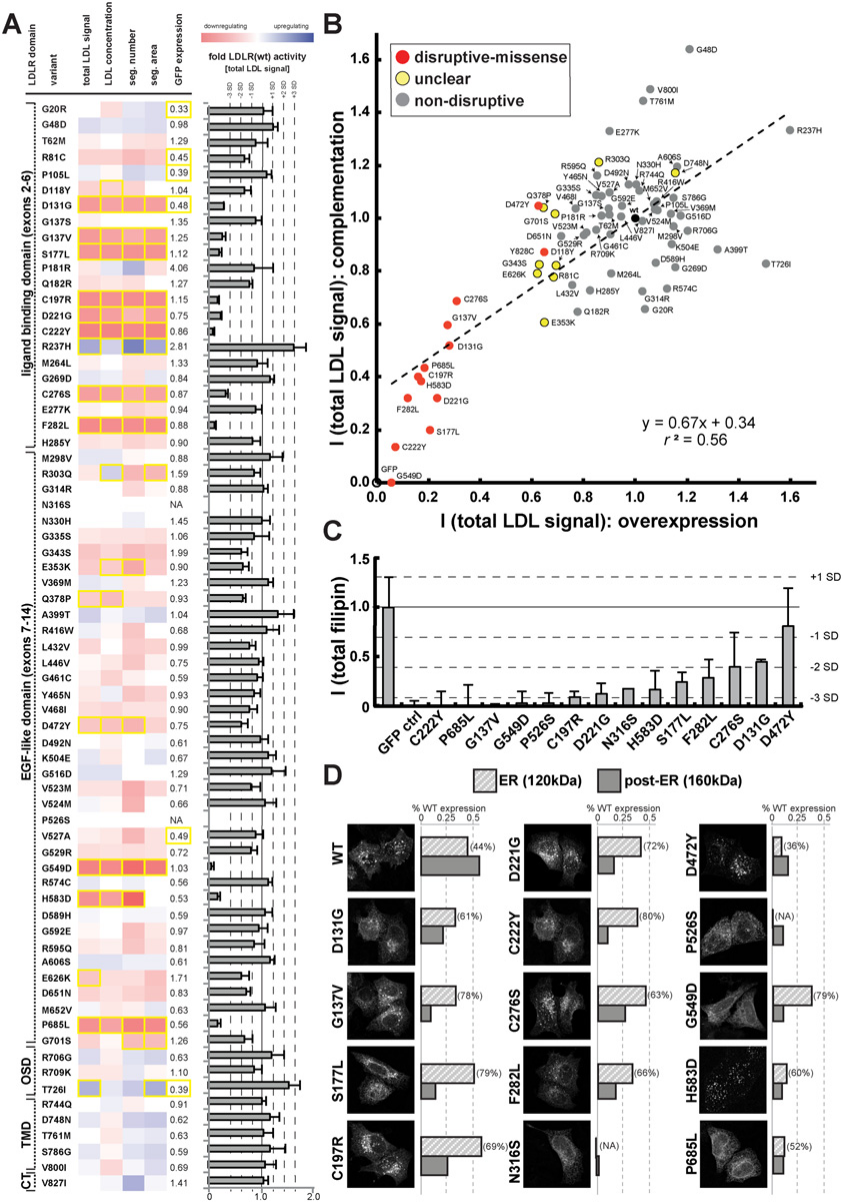
Cell-based functional profiling distinguishes disruptive from non-disruptive rare missense variants in the low-density lipoprotein receptor (*LDLR*) gene as identified through exome sequencing of 3,325 individuals. **(A)** Rare *LDLR* missense variants from exome sequencing of the ATVB cohort were individually introduced into LDLR’-GFP, transiently expressed in HeLa-cells, and the impact on cellular uptake of fluorescently-labeled LDL (DiI-LDL) was quantified for the indicated four parameters and GFP-expression. Shown are means from 3–4 independent experiments per variant relative to wild-type LDLR’-GFP. Phenotypes (red, reducing; blue, increasing) meeting statistical criteria (p<0.05; deviation >1) are framed in orange. Variants that significantly reduced LDL-uptake in ≥3 DiI-LDL parameters, including total LDL signal, were classified as “disruptive-missense” (for details, see Methods). OSD, O-linked sugars domain (exon15); TMD, transmembrane domain (exons16–17); CT, carboxy-terminus (exon18). **(B)** Comparison of mean total DiI-LDL signal intensities within endosome-like intracellular compartments (“total LDL signal”) between the overexpression setting (ΔLDLR’-GFP only) and a complementation setting (siRNA against endogenous LDLR together with ΔLDLR’-GFP) relative to wildtype LDLR’-GFP (wt, black circle) and GFP control (GFP, open circle). Variants classified as “non-disruptive” failed to reach significance in any parameter under neither experimental setting. **(C)** For 14 variants classified as disruptive-missense, impact upon overexpression on cellular levels of free cholesterol (FC) was determined. Shown are means±s.d. relative to wildtype LDLR’-GFP from 2–4 independent experiments. **(D)** Determination of subcellular localization of LDLR’-GFP disruptive-missense variants. Shown are maximal projections of confocal stacks of representative cells transiently transfected with indicated mutants. Bar graphs reflect ΔLDLR’-GFP levels on Western Blots (shown in S5 Fig.; means from 2 experiments) of endoplasmic reticulum (ER; 120kDa) relative to post-ER (160kDa) form of the LDLR protein relative to total wildtype LDLR as determined by ratiometric measurements. For each mutant, contribution of ER- relative to total LDLR’-GFP protein are indicated in percent.

We applied this workflow to systematically test which of the rare *LDLR* missense alleles revealed by exome sequencing of our large population cohort disrupted LDLR function. Systematic experimental analyses of LDL-uptake into cells assigned each missense variant a distinct phenotypic profile that enabled conclusions on its mechanisms (Fig. 3A; S4 Fig. and S1 Spreadsheet). Results from overexpression and complementation correlated well (for instance, *r* ^2^ = 0.56 for parameter “total LDL signal”; Fig. 3B; S4 Table), thus validating most of each other’s findings. Overall, 14 missense variants strongly inhibited LDLR function, typically by reducing LDL-uptake to 6–31% of the wildtype receptor, and were classified as “disruptive-missense”. As an independent validation, we measured whether these variants also impacted total cellular levels of free cholesterol, another phenotype that we have previously shown to vary dependent on LDLR activity [22]. Indeed, all but one disruptive-missense variant not only reduced LDL-uptake, but also free cholesterol levels to less than 50% of controls (Fig. 3C; S5 Table). The only non-validated disruptive-missense variant p.D472Y, as well as two transport-inhibiting ER-associated mutants (p.N316S; p.P526S) reduced LDLR’-GFP protein expression, which indicated an impact on either LDLR biosynthesis or turnover. Like most known FH mutants [18] the majority of disruptive-missense variants clustered in the apoB-ligand binding domain of LDLR and was completely or partially retained in ER-like membranes (Fig. 3D; S5 Fig.). Another 10 variants were defined as of “unclear” functional significance, as they met some, but not all required significance criteria (see Methods). The remaining 46 variants were classified as “non-disruptive”.

### Carriers of *LDLR* alleles identified as disruptive-missense have higher plasma LDL-C

**Figure 4:**
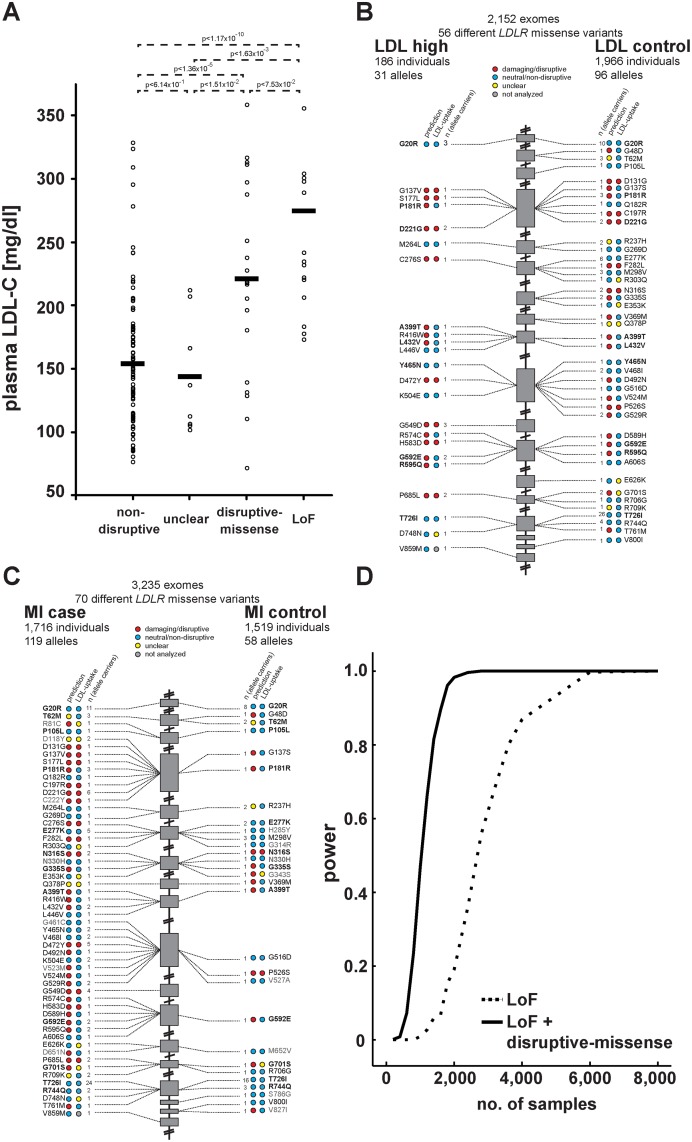
Functions and distribution of *LDLR* rare missense alleles identified through exome sequencing of 3,235 individuals. **(A)** Plasma LDL-C (in mg/dl) in *LDLR* missense allele carriers (dots) from the ATVB cohort according to functional category (for classification, see Methods). LoF, loss-of-function. Means are indicated by horizontal bars. p-value was determined by 2-sided, 2-tailed Student’s t-test. **(B, C)** Individual *LDLR* missense variants identified through exome sequencing of indicated number of individuals are depicted according to genomic position starting at the 5’end (top). The numbers next to each variant represent the number of times the respective variant was observed in cases and controls, respectively, with regard to plasma LDL-C levels (b) and early-onset myocardial infarction (MI; c). Colors in circles represent indicated functional classes as determined either by an overlap of four bioinformatic prediction tools (PolyPhen-2, SIFT, MutationAssessor and MutationTaster; see Methods) (“prediction”) or cell-based experimental studies of LDL-uptake. Variants in bold have been observed in both, cases and controls. **(D)** Power calculations for the number of sequenced individuals needed to reach exome-wide significance (p<2.5×10^-6^, reflected by power = 1) for association with MI-risk when the indicated classes of rare *LDLR* alleles are taken into account. For details, see Methods.

We next compared our *in vitro* results to plasma LDL-C levels available for 2,152 of the individuals in our studied cohort. For 20 variants previously listed in four *LDLR* locus-specific databases as either causing FH or neutral, experimental data matched with clinical interpretation in 95% of cases (S6 Table and Methods). Importantly, plasma LDL-C was significantly higher in disruptive-missense (221mg/dl) than in non-disruptive (154mg/dl; p<1.36×10^-5^) and intermediary to LoF *LDLR* allele carriers (275mg/dl) (Fig. 4A; relative to 135mg/dl in individuals with two wild-type *LDLR* alleles [12]). As discussed further below, only few carriers of a respective variant class showed LDL-C levels outside the expected range.

### Considering *in vitro* data for rare-variant association testing refines the risk of *LDLR* allele carriers for high LDL-C and MI by orders of magnitude

These results demonstrated that our strategy efficiently enriched for FH alleles and suggested that considering experimental data might also enhance rare-variant association testing. For this, disruptive-missense alleles were enumerated in cases and controls across the entire cohort (Fig. 4B,C) and tested for association with LDL-C and MI. Indeed, collapsing only disruptive-missense (instead of all *LDLR* missense) alleles strongly increased odds ratios from 3.2 to 18.6 for association with LDL-C, and from 1.9 to 12.1 for association with MI-risk (Table 1, Tables S2–3). Enumerating disruptive-missense together with LoF variants firmly established rare variation in *LDLR* as associated with plasma LDL-C (p<6×10^-19^; OR = 25.3) and MI-risk (p<2×10^-10^; OR = 20.0) on the population level. Consistent with a theoretically predicted 2.2- to 3-fold reduction in the number of samples needed to be sequenced [10], power simulations suggested that through integration of experimental data sequencing of only 1,200–1,400 (instead of 3,000–4,000) cases and controls would be sufficient to associate rare variation in *LDLR* with MI-risk at genome-wide significance (Fig. 4D). Notably, experimental data empowered RVAS considerably more than functional prediction tools that correctly evaluated all 14 disruptive-missense variants as damaging, yet consistent with previous observations [24] showed higher type-I-error rates (Table 1; S1 Table; S7 Table and Methods).

### For individual low-frequency *LDLR* alleles, effects on plasma LDL-C and cellular LDL-uptake correlate

**Figure 5:**
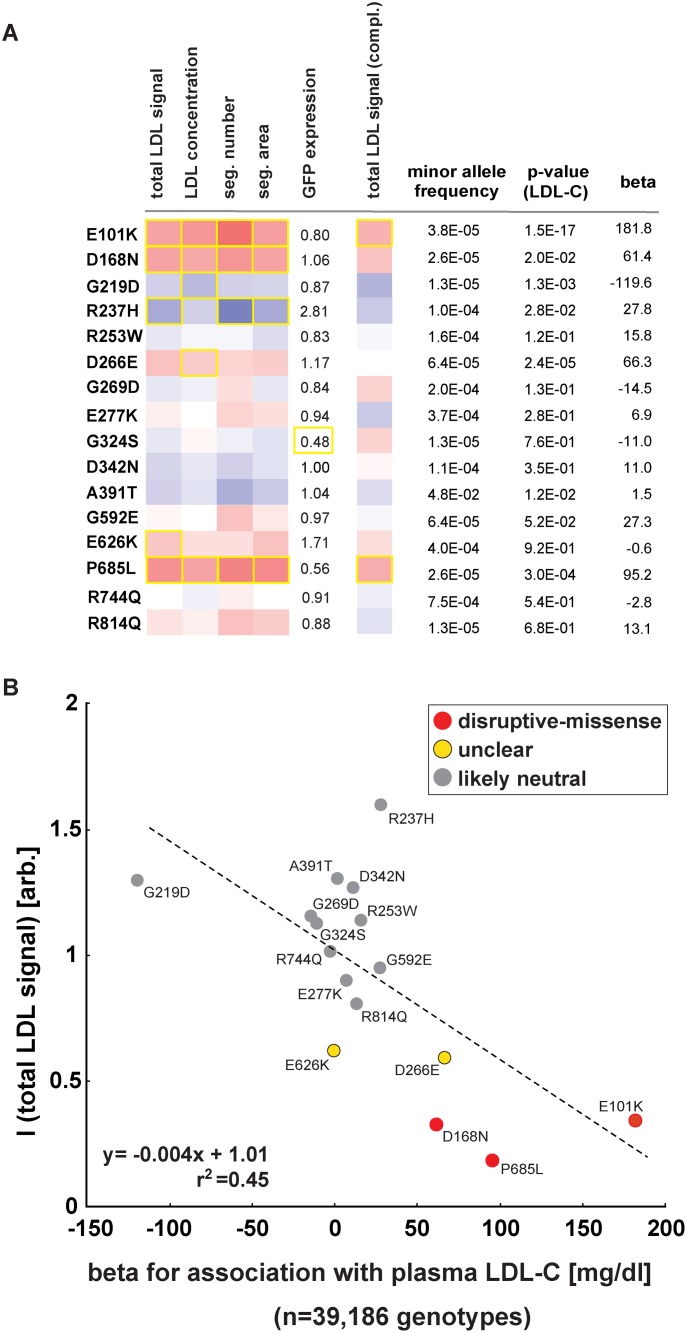
Impact of individual *LDLR* missense variants on cellular LDL-uptake correlates with single-variant association results for plasma LDL-C in ~40,000 individuals. **(A, B)** Comparison of results from cell-based functional profiling of LDL-uptake (overexpression and complementation) and single variant association tests for 16 *LDLR* missense variants represented on the exome-chip and identified by genotyping 39,186 individuals from multiple cohorts. Shown are means from 3–4 independent experiments per variant relative to wild-type LDLR’-GFP. Phenotypes (red, reducing; blue, increasing) meeting statistical criteria as described in Methods are framed in orange. Effect size (beta) is provided as the difference of means of plasma LDL-C levels between variant carriers and non-carriers (in mg/dl).

Most missense alleles identified in sequencing studies are rare. At limited sample sizes RVAS thus typically fall short on clarifying by how much any individual rare variant contributes to a complex trait [10]. Conversely, one advantage of *in vitro* studies is that once a variant has been observed in a population, variant frequencies do not matter. We aimed to test whether experimental data could support genetics also for single variant association analyses. In order to increase the number of observations per variant, we analyzed the function of 16 *LDLR* missense alleles that are represented on the Illumina HumanExome v1.0 SNP array (“exome-chip”) and that were genotyped in 39,186 individuals characterized for LDL-C (Fig. 5; S7 Table and S1 Spreadsheet) [25]. Overall, effect sizes between genotyping and *in vitro* experiments correlated well (*r* ^2^ = 0.45). Importantly, the variants with the highest beta (p.E101K, p.P685L) most pronouncedly inhibited LDL-uptake in cells, supporting our hypothesis that systematic experimental data will not only be informative for gene-burden analyses, but also in clarifying by how much individual rare and low-frequency variants contribute to genetic etiologies.

## Discussion

Our study demonstrates that distinguishing disruptive from non-disruptive missense alleles in a well-described disease gene (*LDLR*) through systematic functional characterization *in vitro* can further our understanding how rare, potentially damaging genetic variation contributes to common, complex (hypercholesterolemia; MI) as well as Mendelian disease (FH). Thus far, the role of cell-based experiments in human genetics has either been to validate assumed associations between one to few variants and disease, or to better comprehend the mechanisms why variants firmly identified through genetics are pathogenic [2]. Conversely, our study, together with few previous studies [24, 26, 27], predicts that soon unbiased experiments will attain a much more central role in human genetics that could extend to the very core of disease gene discovery.

Optimizing RVAS by stratifying missense alleles according to their *in vitro* ascertained functions may prove especially powerful to identify and validate genes under a high selective pressure where disruptive-missense are swamped by neutral alleles and sample sizes needed for association become enormous [10]. For *LDLR*, as a gene with an average endogenous mutation rate, Zuk et al. [10] estimated 17% of missense variants as being disruptive, which is well in line with the 20% we identified experimentally. On the other hand, our strategy may be less amenable to very essential genes where modulation of cellular levels by overexpression or knockdown is less well tolerated. Also, sensitivity of our approach may be limited for genes where the correlation between measured phenotype and gene function is less direct than between LDLR levels and LDL-uptake, or where the odds ratios of even disruptive alleles are small.

For *LDLR*, our binary classification of alleles as either disruptive or non-disruptive simplifies the range of functional consequences that missense variants can exert on receptor activities [16, 18]. For instance, the inclusion of only disruptive variants for association testing neglects hypomorphic variants that reduce LDLR activity by only few percent. In our study, this is evidenced by slightly elevated odds ratios also in non-disruptive allele carriers. It thus can be expected that through segregation analyses in families, or through more sensitive *in vitro* readouts, several such alleles will be identified as FH mutants in the future. Although the individual effect of hypomorphic alleles on LDLR activity may be small and, consistent with previous assumptions [10], they in sum add only little power to association tests, future RVAS may profit from counting in also hypomorphic alleles in form of adjusted functional weights.

An intriguing hypothesis is that in addition to rare variation in *LDLR*, further genetic or environmental factors contribute to increase LDL-C in some carriers of alleles that in our experiments scored as non-disruptive. However, a thorough analysis of known common and rare genetic risk factors from the exomes of 23 individuals with plasma LDL-C levels that did not match expectations from our *in vitro* analyses did not reveal clear evidence for epistatic effects (see paragraph Search for reasons of aberrant LDL-C in LDLR missense allele carriers in Methods). More carriers of the identical rare alleles, or an even stronger relationship between genetic variant, intermediate and clinical phenotype than between LDLR, LDL-C and MI are needed to exploit the full spectrum of information available from large-scale sequencing studies. Moreover, relationships between *in vitro* ascertained function and *in vivo* phenotypes are likely to improve further when the analyzed cohorts can be stratified for important confounders, here, for instance, intake of LDL-lowering medications [28], which was unavailable for this study.

For Mendelian genetics it is worthwhile to note that seven of the variants analyzed here have recently been observed incidentally through clinical exome sequencing of individuals [29, 30] and are listed as potentially requiring medical intervention [20]. Interestingly, however, based on our *in vitro* studies none of these variants is a strong candidate for causing FH. A more comprehensive annotation of important disease genes through studies like ours together with family-based segregation analyses may help to considerably precise health risks in the future. Through generating scalable cell-based assays for relevant intermediate phenotypes and statistical tools that better incorporate genetic with heterogeneous functional datasets, we expect that composite sequencing-biological studies will become invaluable to human genetics in order to face the flood of novel variants from the ever increasing number of sequenced genomes.

## Materials and Methods

### Genetics analyses

**Study cohorts**. The Italian Genetic Study of Early-onset Myocardial Infarction (ATVB) is a European case-control collection designed to study the genetics of MI-susceptibility [12, 31, 32]. Exome-sequenced MI cases (n = 1,716) include survivors of a first acute myocardial infarction (defined as more than 30min resting chest pain accompanied by typical ECG and serum abnormalities) at an age of less than 46 years with angiographically documented coronary artery disease. Exome-sequenced MI controls (n = 1,519) were matched for age, sex, and geographical origin and assessed for further MI-risk factors (S10 Table). Principle component analyses did not indicate selection bias between cases and controls (S7 Fig.). For 2,152 subjects (66.5%), plasma low-density lipoprotein cholesterol (LDL-C) at enrollment was available, among them 1,184 MI cases and 968 MI controls. Overall, 251 subjects showed hypercholesterolemia defined as LDL-C above 190mg/dl (4.91mmol/l) (LDL cases) and according to Simon Broome criteria [19, 33] a high likelihood for FH. For 1,901 subjects LDL-C was in the normal range or only moderately elevated (<190mg/dl; LDL controls). As expected, high LDL-C was strongly associated with increased MI-risk in this cohort [12].

Genotype data were obtained from a meta-analysis of 39,186 independent samples characterized with the Illumina HumanExome v1.0 SNP array (“exome-chip”). Samples were from individuals of European ancestry derived from 25 studies on the impact of rare and low-frequency coding variation on plasma lipids [25].

**Ethics statement**. All analyses in this study conformed to the ethical guidelines of the 1975 Declaration of Helsinki in its crespective latest version. The study has been approved by an IRB from the Broad Institute under protocol number 2013P001840.

**Exome sequencing and exome-chip genotyping**. Exome sequencing was performed at the Broad Institute Genomics Platform as described previously [34]. Details on all specific steps for reliable variant calling from raw sequence or exome-chip data, as well as performed quality controls for the cohorts used in our study are provided in Do et al. [12] and Peloso et al. [25].

***LDLR* gene variant selection**. *LDLR* nomenclature throughout the manuscript relates to *Homo sapiens* low density lipoprotein receptor (*LDLR*) transcript variant 1 (NM_000527.4; ENST00000558518/ Ensembl73) encoding a protein of 860 amino acids. Overall, 79 DNA sequence variants in *LDLR* were functionally characterized in this study (S7 Table and S1 Spreadsheet) out of which 78 were identified through exome sequencing and/or exome-chip profiling and one (p.Y828C) was selected from the literature. Based on available biochemical and clinical information, two FH-mutants with firmly established pathogenic mechanisms were chosen as controls, p.G549D [FH Genoa] as example for a transport-inhibiting (class-2) mutant [18] and p.Y828C [FH JD-Bari] that prevents association of LDLR with clathrin-coated pits and its internalization into the endosomal system (class-4) [18, 35]. Exome sequencing of the ATVB cohort [12] identified a total of 82 rare coding variants in *LDLR*, distributing on 194 alleles. Of these variants, 12 were clear loss-of-function (LoF), causing in 8 cases introduction of a preterm stop codon (p.Q33*; p.Q102*; p.E140*; p.C155*; p.R350*; p.Y419*; p.W533*; p.Q770*) and in 4 cases disruption of splice-donor sites (19:11213463_G/A; 19:11224126_G/A; 19:11224439_G/A; 19:11227676_T/C; NCBI37). Consistent with markedly reduced LDLR activity, LoF variants strongly associated with plasma LDL-C (Table 1; Fig. 4A; S3 Table and [12]) and were omitted from cell-based studies. All 70 ATVB *LDLR* missense variants were selected for *in vitro* functional characterization, and 69 comprehensively profiled as described below (with the exception of p.V859M that due to its localization at the LDLR carboxy-terminus failed repetitive cloning attempts). Forty-three (61%) of these missense variants were present only once among the 6,470 ATVB chromosomes, corresponding to a minor allele frequency (MAF) of 1.5×10^-4^. Twenty-five variants occurred in 2–7 study participants, and two variants in 19 and 40 subjects, respectively (S1A Fig.). Apart from p.T726I with a MAF of 0.00618, all variants fulfilled our definition of being rare by showing a MAF of less than 0.005, corresponding to one heterozygote carrier per 100 study participants. *LDLR* variants identified in the ATVB cohort were complemented by 16 variants represented on the Illumina HumanExome vs1.0 SNP array that were identified by genotyping 39,186 European subjects from diverse studies characterized for plasma LDL-C [25]. Seven variants (p.R237H; p.G269D; p.E277K; p.G592E; p.E626K; p.P685L; p.R744Q) overlapped between both studies. Frequency distributions of *LDLR* coding variants among participants of the NHLBI exome sequencing project (ESP) (6,823 individuals; 13,646 chromosomes) (S1B Fig.) were downloaded from the Exome Variant Server (http://evs.gs.washington.edu/EVS/; accessed October 2014).

**Locus-specific *a priori* information**. For all 79 variants that underwent functional characterization in this study we systematically searched for availability of *a priori* clinical or functional information. For this, four public databases retaining locus-specific information on variation in *LDLR* were queried: the Universal LDLR mutation database (http://www.umd.be/LDLR/) [36]; the *LDLR* LOVD database at University College London (http://www.ucl.ac.uk/ldlr/) [37]; the NCBI ClinVar database (http://www.ncbi.nlm.nih.gov/clinvar) [38]; and the Human Gene Mutation Database (professional version) (www.hgmd.org) [39]. Information from 111 publications that these databases referred to (S6 Table and Supplemental References) allowed us to classify 19 *LDLR* variants as either previously validated FH mutant (n = 7), likely benign (n = 5), or of unclear disease relevance (n = 7; including variants identified in compound-heterozygous individuals in combination with a clear FH mutation). All but one FH mutant (p.V523M [FH-Kuwait] that in homozygous fibroblasts was reported as associated with 12–25% residual LDLR activity [40]) met our criteria for being “disruptive-missense” (see below). Except for one variant (p.D118Y) for which disease relevance also after *in vitro* functional testing remained unclear, all other previously observed variants were classified as non-disruptive. Of four additional variants that in the LDLR LOVD database were listed as FH, but that had not previously been validated *in vitro*, only one variant (p.C222Y) met our criteria as disruptive-missense. Of 56 variants that were listed in HGMD with the phenotype hypercholesterolemia, yet without functional evidence for this, our analyses classified 13 as disruptive-missense.

**Comparison to bioinformatics prediction tools**. For each missense variant we determined its likelihood to interfere with LDLR protein activity by applying four commonly used *in silico* functional predicition tools under default settings: PolyPhen-2 (http://genetics.bwh.harvard.edu/pph2) [41], SIFT (http://sift.jcvi.org) [42], MutationAssessor (http://mutationassessor.org) [43] and MutationTaster (http://www.mutationtaster.org) [44] (S1 Table). Different result categories of each algorithm were assigned distinct numerical values (PolyPhen-2: damaging/probably damaging,-1; possibly damaging,0; benign,+1; SIFT: damaging,-1; tolerated,+1; MutationAssesor: high/medium:,-1; neutral/low,+1; MutationTaster: disease-causing,-1; polymorphism,+1). A summed composite score was calculated for each variant from the overlap of all four prediction tools. A composite score of more than 1 was considered as likely benign, of 0 as unclear and of less than-1 as likely FH. Overall, bioinformatics prediction tools classified 40 of the 79 studied LDLR missense variants (51%) as FH-like, 7 (9%) as of unclear disease relevance and 32 (40%) as likely benign (S7 Table).

**Association testing**. Rare variant association tests were performed by enumerating all rare *LDLR* alleles of a distinct class (clear LoF; all missense; bioinformatically predicted as damaging; disruptive-missense; non-disruptive; and unclear) and by calculating association of the burden of variants in cases and controls with plasma LDL-C and MI using Fisher’s exact test (see also [12]) (Table 1, S2 Table). To estimate effect sizes (beta) for continuous levels of LDL-C in the ATVB cohort (S3 Table), linear regression analysis was performed with LDL-C (in mg/dl) as outcome variable, carrier status as independent variable, and sex and age as covariates.

**Power calculations for *LDLR* rare variant association with MI**. Based on sequence data from 3,325 ATVB participants, we performed sample size extrapolations for association signals driven by the burden of rare *LDLR* variants of either LoF variant carriers alone, or LoF variant carriers combined with carriers of variants identified as disruptive-missense. The relative risk of a mutation carrier was assumed to be 5.0. Prevalence of MI was assumed as 0.05. Case:Control ratio was assumed as 1. The number of rare variants was extrapolated into 500,000 individuals. One thousand simulations were performed at a given sample size with intervals of 200 samples (from n = 0–2,000), 400 samples (from n = 2,000–4,000) and 2,000 samples (from n = 4,000–20,000). Power reflects the percentage of simulations that reached genome-wide significance (set at 2.5×10^-6^ to account for testing of ~20,000 genes) at a given number of samples.

### Cell-based functional analyses

**Cells and reagents**. HeLa-Kyoto cells and their suitability for measuring the dynamics of LDL-uptake and cellular levels and distribution of free cholesterol (FC) were described in our previous studies [21, 22]. DiI-LDL (Life Technologies), Filipin III (Sigma), Draq5 (Biostatus), Dapi (Hoechst), 2-hydroxy-propyl-beta-cyclodextrin (HPCD) (Sigma), Lipofectamine 2000 (Invitrogen) and Oligofectamine (Invitrogen) were purchased from the respective suppliers.

**cDNA cloning, siRNAs and site-directed mutagenesis**. A sequence-verified cDNA-clone encoding full-length human LDLR carboxy-terminally linked to EGFP was described previously to adequately reflect activities of the wild-type receptor [22]. To guarantee knock-down of the mRNA encoding the endogenous receptor, but not the heterologously expressed LDLR-GFP cDNA during complementation experiments, three silent mutations (c.A1053G, c.C1056T and c.A1059G) were introduced at Wobble-bases within the 19-nucleotide consensus sequence (CAGCGAAGATGCGAAGATA) of LDLR-siRNA s224006 (Applied Biosciences) by site-directed mutagenesis (see below) using the following primer sequences: 5'-ctggtggcccagcgaaggtgtgaggatatcgatgagtgtca-3' (forward) and 5'-tgacactcatcgatatcctcacaccttcgctgggccaccag-3' (reverse). LDLR-siRNA efficiently reduced levels of the endogenous LDLR mRNA by ~30% and of the endogenous protein by ~75%, respectively, significantly reduced cellular LDL-uptake [22] and abrogated expression of LDLR-GFP. In contrast, levels of the siRNA-resistant LDLR-GFP construct (termed LDLR’-GFP) were unaffected by siRNA-treatment (S2A Fig. and [22]). Subcellular distribution and effect upon overexpression and complementation on DiI-LDL uptake were indistinguishable between LDLR-GFP and LDLR’-GFP (Fig. 2A; S2B Fig. and [22]). LDLR’-GFP served as a template for introduction of studied missense variants using QuikChange Lightning Site-directed mutagenesis kit (Agilent) according to the manufacturer’s instructions. Oligonucleotides for generating distinct *LDLR* variants were designed using the QuikChange Primer design tool (Agilent), ordered from Metabion (Martinsried, Germany) and are listed in S11 Table. During complementation experiments, siRNA s229174 (Silencer Select, Applied Biosystems) served as a non-silencing control siRNA.

**Overexpression, complementation and biological assays**. For overexpression analyses, cells were seeded on glass coverslips in 12-well plates (Corning) at a density of 4×10^4^ cells/well, cultured in DMEM (PAA)/2mM L-glutamine/10% FBS (Biochrom) for 24h at 37°C/5% CO_2_, and fluid-phase transfected with 2μg cDNA/well using Lipofectamine2000 (Invitrogen) according to manufacturer’s instructions. Assays to monitor cellular uptake of fluorescently-labelled LDL (DiI-LDL) were performed as described in more detail in a previous publication [21]. In brief, cells cultured in serum-free medium and exposed to 1% 2-hydroxy-propyl-beta-cyclodextrin for 45min were labelled with 50μg/ml DiI-LDL (Invitrogen) for 30min at 4°C. DiI-LDL uptake was stimulated for 20min at 37.5°C before washing off non-internalized dye for 1min in acidic (pH 3.5) medium at 4°C, fixation, and counterstaining for nuclei (Dapi, Draq5) and cell outlines (Draq5). For quantification of cellular cholesterol, cells were stained with 50μg/ml Filipin III in PBS (from a stock-solution of 1mg/ml in di-methyl-formamide), fixed, and counterstained with cell and nuclear marker Draq5. For complementation experiments, cells were seeded at an identical density, cultured in DMEM (PAA)/2mM L-glutamine/10% FBS (Biochrom) for 24h at 37°C/5% CO_2_, and fluid-phase transfected with 0.5μl/well of 30μM LDLR-siRNA (s224006) or non-silencing control siRNA (s229174) for 24h using Oligofectamine according to manufacturer’s instructions. One day after siRNA transfection, cells were co-transfected with GFP-cDNAs using Lipofectamine2000 as described above, and cultured for another 24h before biological assays were performed and samples were prepared for microscopic analysis. Overexpression experiments were performed in 3–5, rescue experiments in 1–6 biological replicates per variant. Images were acquired automatically with identical baseline settings from 30 different positions/sample on an Olympus IX81 automated microscope using an UPlanApo 20×0,7NA objective and ScanR software vs. 2.1.0.15 (Olympus Biosciences).

**Image data analysis**. All images were visually quality controlled using Image J 1.46r (Wayne Rasband, National Institutes of Health, Bethesda) in order to exclude pictures of insufficient technical or biological quality (e.g., due to image acquisition out of focus or aberrant cell density). Biological replicates for each variant analyzed were compared to several controls present during each individual experiment. Each overexpression experiment included wild-type LDLR’-GFP as a positive control as well as two negative controls, i.) a sample where cells expressed a construct encoding only EGFP without the receptor protein (“GFP-control”) and ii.) a sample where cells were exposed only to transfection reagents, but not cDNA (“transfection-control”). Each complementation experiment included four controls: cells transfected either with i.) LDLR siRNA or ii.) negative control siRNA, but no cDNA, as well as two samples where LDLR siRNA-treated cells were co-transfected with either iii.) LDLR’-GFP or iv.) GFP-control cDNAs. Images were analyzed with customized pipelines based on Cellprofiler 2.0 software (http://www.cellprofiler.org) [45]. Analysis strategy was adjusted from [22] and is outlined in S3 Fig. In brief, outlines of individual cells were approximated by stepwise dilation of masks generated from images of Draq5 and/or Dapi (for LDL-uptake) stained cell nuclei. Mean cellular GFP signal (“GFP-expression”) was quantified from background-subtracted images within areas defined as cells. Filipin (for FC) or DiI-signal (for LDL-uptake) was quantified from background-subtracted images within masks that reflected distinct intracellular compartments resembling endosomes (for LDL-uptake) or lysosomes (for FC: see also [22]) as identified by local adaptive thresholding. When cells or compartments exceeded a range of pre-defined parameters (such as signal intensity or shape, minimal/maximal diameter, minimum allowed distance to neighbouring mask or edge of the image) they were omitted from further analysis to exclude for instance dividing or apoptotic cells. Mean cellular background intensity in the GFP channel was determined from the transfection-control sample of each experiment. Tabulated numeric results from image analyses were further processed with customized R-pipelines (R-Studio Inc. vs 0.97.336). Cells with GFP-intensities beneath the 97 percentile of this transfection control sample were defined as “GFP-negative”, and this threshold was applied to determine GFP-negative cells also from the other samples of a respective experiment. Conversely, cells were defined as GFP-expressing (“GFP-positive”) if cellular GFP-signals exceeded this GFP-negative threshold by at least two-fold. Complementation experiments were performed under a “rescue”, but not overexpression setting. Specifically, an upper threshold was introduced for the Cy3 (DiI)-channel, and DiI-LDL uptake was quantified only from the fraction of GFP-positive cells that showed less than 1.25-fold the mean “total LDL signal” (see below) of cells in the transfection-control sample, or less than 5 times the mean “total LDL signal” of cells treated with LDLR siRNA without concomitant cDNA transfection, or cells co-transfected with LDLR-siRNA and GFP-control plasmid, respectively. A justification for this upper threshold is provided by complementation experiments shown in S2B Fig. that demonstrate that reduced DiI-LDL uptake in response to LDLR knockdown can be fully complemented by co-expressing wild-type LDLR’-GFP at only 10–20% of its maximal expression level. For LDL uptake experiments five parameters were quantified per cell: (i) total DiI signal intensity within intracellular endosome-like segments (“total LDL signal”), (ii) mean DiI signal intensity within segments per cell (“LDL concentration”), (iii) number of individual segments within cell masks (“seg. number”), (iv) summed area of all segments within cell masks (“seg. area”), and (v) mean cellular GFP signal intensity (“GFP-expression”).

**Statistical analysis of imaging data**. For each parameter, means were calculated from all cells per image, and cells were classified as either GFP-positive or GFP-negative. Results from different images of the same biological replicate were averaged, and the ratios of GFP-positive relative to GFP-negative cells were determined. A minimum of 25 GFP-positive cells per variant was required to be considered as independent experimental replicate. Results from different biological replicates were then averaged and compared to outcomes for LDLR’-GFP. Impact of a variant on a distinct parameter was considered as significantly different from wildtype LDLR’-GFP when a paired, two-tailed Student’s t-test resulted in p-values of less than 0.05 and a “deviation value” (a z-score-like measure described in detail in [22]) for parameter total LDL-signal was larger than 1. A variant was categorized as “disruptive-missense” (i.e., severely disrupting LDLR activity as would be expected from an LoF-mutant) if under the overexpression setting “total LDL signal” as well as at least two other parameters reached significance. Under the complementation setting, significance in the parameter “total LDL signal” was regarded as sufficient to validate a variant identified as “disruptive-missense” under the overexpression setting. In order to be classified as “non-disruptive”, none of the eight DiI-LDL parameters quantified from overexpression and complementation settings was allowed to reach significance. A variant was classified as of “unclear” functional significance if it met neither criteria for category “disruptive-missense” nor “non-disruptive”. To test for possible interdependence of measured four DiI-LDL parameters, pairwise Pearson’s correlation values were calculated across the entire dataset (comprising 79 different variants plus wildtype LDLR’-GFP; S7 Table). Consistent with our expectations and the literature (see also [22]), parameters “total LDL signal”, “LDL concentration”, “seg. number” and “seg. area” correlated well, both among each other as well as between overexpression and complementation settings, reflecting a high reproducibility of individual results (S4 Table).

For measuring the impact of disruptive-missense variants on free cholesterol (FC) levels, total filipin signal intensities from lysosome-like intracellular areas were quantified as described [22] from cells cultured and analysed in 96-well plates. Variants that significantly affected cellular FC were determined from the ratio of signal intensities in GFP-positive relative to GFP-negative cells according to identical significance criteria as described above (apart from p.N316S for which no significance could be determined as it reached the minimal number of required GFP-positive cells in only one out of four biological replicates).

### Secondary experimental analyses

**Determination of LDLR protein levels**. For quantification of LDLR protein levels by Western Blot (Fig. 3D, S2 and S5 Figs.), HeLa-Kyoto cells co-transfected with cDNAs and siRNAs as described above were lysed in 40μl SDS-loading buffer and subjected to immunoblotting with anti-LDLR (Cayman Chemicals), anti-GFP (Roche) and anti-actin (Sigma). Signal intensities of lanes representing 120kDa and 160kDa isoforms of LDLR protein were quantified from background subtracted images using Image J 1.46r (Wayne Rasband, National Institutes of Health, Bethesda) and normalized to levels of beta-actin.

**Determination of ΔLDLR’-GFP subcellular localization**. Subcellular localization of LDLR’-GFP variants identified as disruptive-missense were re-analyzed at higher resolution using a Zeiss LSM780 laser-scanning confocal microscope using a 63x objective. Assignment of individual variants to different FH-mutant classes was based on i.) phenotypic effects on DiI-LDL uptake, ii.) GFP expression level; and iii.) degree of localization to endoplasmatic reticulum-like relative to endosome-like structures or the plasma membrane as determined visually.

### Search for reasons of aberrant LDL-C in LDLR missense allele carriers

Twenty-three *LDLR* missense allele carriers from the exome-sequenced cohort (Fig. 1) showed plasma LDL-C levels that did not match expectations from *in vitro* analyses. For instance, in five carriers of disruptive-missense alleles that all showed early-onset MI, LDL-C was below 190mg/dl. Besides the unlikely possibility for reduced penetrance of heterozygous FH [46] and MI for other causes, a reasonable explanation for this could be that these individuals received LDL-lowering therapies (e.g., statins) at study inclusion. As this information was unavailable to us, precision of the type I error rate for our cell-based analyses is difficult, although it can be assumed as likely small. Of higher relevance is why some carriers of LDLR alleles classified as non-disruptive still showed elevated plasma LDL-C and/or MI, although this is in part this justified by the use of strict sensitivity thresholds that excluded potentially hypomorphic variants from association testing (see Discussion).

It is tempting to speculate that additional genetic variants could have their share in increasing LDL-C in some non-disruptive *LDLR* allele carriers. One reason for this could be compound-heterozygosity for more than one rare variant at the *LDLR* locus. For instance, we identified one carrier of the most likely neutral variant p.G20R as also carrying the FH mutant p.G549D, and the latter variant is much more likely to explain that individual’s plasma LDL-C of 218mg/dl. Likewise, compound-heterozygosity for two hypomorphic variants could impair receptor activities in the range of a classic FH-mutant. This is best exemplified by another ATVB individual compound-heterozygous for neutral variants p.L432V and p.Y465N and LDL-C of 309.4mg/dl.

Also, increasing evidence supports a di- or polygenic contribution to the regulation of plasma lipid levels and MI-risk [47–49], and alterations in other genes might explain elevated LDL-C in non-disruptive allele carriers, or unexpectedly low LDL-C in disruptive allele carriers. To test the hypothesis that common risk variants might modify LDL-C levels in these individuals, we calculated polygenic risk scores for variation in LDL-C according to [48] based on 20 lead SNPs from genome-wide association studies for plasma lipids [47] that were represented on the exome chip (S8 Table). Exome chip genotypes were available for 2,433 ATVB study participants. Risk scores relative to plasma LDL-C for all participants are plotted in S6 Fig. In the 23 individuals with unexpectedly low or high LDL-C we did not observe a major contribution of 20 common risk variants when this subcohort was compared to the rest of the ATVB cohort.

We also analyzed these 23 individuals for the presence of rare coding variation in 12 further genes linked to Mendelian causes of abnormal plasma LDL-C (*ABCG5*, *ABCG8*, *ANGPTL3*, *APOA5*, *APOB*, *APOC3*, *APOE*, *LDLRAP1*, *LIPA*, *MTTP*, *NPC1L1* and *PCSK9*). This produced a total of 21 rare and low-frequency protein-sequence altering variants that distributed over 10 genes (S9 Table). Clinical significance of these variants was evaluated based on information from locus-specific FH databases (for *ABCG5*, *ABCG8*, *APOB*, *LDLRAP1* and *PCSK9*), the Exome Variant Server, ClinVar and HGMD. Only a single variant (p.R238W in *LDLRAP1*) present in a heterozygous state in two of the 23 individuals had previously been reported from patients with autosomal-recessive FH. However, based on an allele frequency of 0.048 in Europeans and because association of this variant with LDL-C across the ATVB cohort, although indicative, does not yet reach genome-wide significance (p<0.00037; Fisher’s exact test), the contribution of this variant to LDL-C levels in the two *LDLR* variant carriers that also carry this *LDLRAP1* variant remains unclear. One rare variant in *APOE* (p.G145D) is described as benign polymorphism. No database or literature data is available on the other 19 variants identified, and none has yet been characterized *in vitro*.

### Supplemental data description

Supplemental Data contains eleven Supplemental Tables, seven Supplemental Figures, one Supplemental Spreadsheet, and Supplemental References.

### Web resources

Exome Variant Server, http://evs.gs.washington.edu/EVS/; Human Gene Mutation Database, http://www.hgmd.org; LDLR UCL LOVD database, http://www.ucl.ac.uk/ldlr/; MutationAssessor, http://mutationassessor.org; MutationTaster, http://www.mutationtaster.org; NCBI ClinVar database, http://www.ncbi.nlm.nih.gov/clinvar; PolyPhen-2, http://genetics.bwh.harvard.edu/pph2/; SIFT, http://sift.jcvi.org; Universal LDLR mutation database, http://www.umd.be/LDLR/

### Accession numbers

Data, including LDLR sequence data and functional annotations, will be available for download from the NCBI ClinVar database (http://www.ncbi.nlm.nih.gov/clinvar/) under accession numbers SCV000189524—SCV000189592 and SCV000189619—SCV000189628.

## Supporting Information

S1 Fig Frequency distribution and predicted mutational effect of *LDLR* missense alleles in the ATVB and NHLBI-ESP cohorts. Shown are frequency distributions of *LDLR* missense alleles discovered through exome sequencing of **(A)** 6,650 chromosomes of the Italian Genetic Study of Early-onset Myocardial Infarction (ATVB) cohort and **(B)** 13,646 chromosomes of the NHLBI exome sequencing project (ESP) study cohort. Mutational impact was estimated from the overlap of four bioinformatic prediction tools as detailed in Methods. Note that 43 out of 70 (61%) variants in ATVB and 48 of 80 (60%) variants in NHLBI-ESP occur in only one single participant of the respective study cohort, and that the number of variants predicted as “damaging” (red) almost equals the number of variants predicted as “neutral” (grey; unclear, yellow).
(EPS)

S2 Fig Efficiency of LDLR knockdown, overexpression and complementation. **(A)** HeLa-Kyoto cells were transfected with indicated GFP-labelled cDNAs and siRNAs as described in Methods and subjected to Westen Blot for GFP, LDLR and beta-actin. Note that LDLR’-GFP encodes for wildtype LDLR protein, but is rendered insensitive to knockdown by LDLR-siRNA through silent mutations at the siRNA-binding site. **(B)** DiI-LDL uptake as reflected by parameter “total LDL signal” (see Methods) in HeLa-Kyoto cells expressing indicated cDNAs and siRNAs. Signal intensities were normalized to cells treated with transfection reagents only. For LDLR’-GFP (blue-shaded columns), quantifications were performed in bins from cells below the indicated upper thresholds (in %) of maximal GFP expression in a sample. In order to exclude cells overcompensating the endogenous LDL-uptake, only those GFP-positive cells were considered for quantifications during the systematic complementation experiments in this study where “total LDL signal” did not exceed an upper threshold of 1.25-fold the mean “total LDL signal” of cells in the “transfection control” samples, or showed less than 5 times the mean “total LDL signal” of cells co-transfected with LDLR-siRNA and GFP-control cDNA (as indicated here by dashed red line). Shown are means±s.d. from 18–25 independent experiments.
(EPS)

S3 Fig Pipeline for automated multi-parametric image analysis of LDL-uptake. Shown are representative images acquired by automated fluorescence microscopy during LDLR variant profiling together with corresponding segmentations generated for image analysis. For quantifying cellular LDL-uptake, GFP-cDNA or control plasmid transfected HeLa-Kyoto cells were exposed to fluorescent DiI-LDL for 20min at 37°C, fixed and stained for cell nuclei (Dapi) or cell outlines (Draq5). Masks representing nuclei, cells and endosome-like compartments were generated using CellProfiler, and DiI-LDL and GFP phenotypic readouts were quantified as detailed in Methods. Bar = 20μm.
(EPS)

S4 Fig Subcellular localization and effect on cellular LDL uptake of *LDLR* missense variants analyzed in this study. HeLa-Kyoto cells expressing LDLR’-GFP constructs carrying indicated variants identified through exome sequencing of the ATVB cohort were cultivated in serum-free medium, exposed to 1% hydroxypropyl-beta-cyclodextrin for 45min, and cellular uptake of DiI-LDL was monitored for 20min at 37.5°C before fixation and preparation for microscopy. Automatically acquired images of randomly selected GFP-positive and neighboring cells are shown for each of the 70 variants studied. Heatmaps indicate means of the four parameters applied to assess LDL-uptake (for details, see S3 Fig. and Methods). Numbers reflect percent of GFP positive cells (“GFP expression”). WT, wildtype LDLR’-GFP. Bar = 15μm.
(EPS)

S5 Fig Disruptive-missense variants reduce LDLR protein levels and shift subcellular distribution towards the endoplasmic reticulum. Lysates of HeLa-Kyoto cells expressing LDLR’-GFP constructs carrying indicated disruptive-missense variants were immunoblotted for EGFP and beta-actin. Blots displayed served for ratiometric measurements of ER- relative to post-ER form of the LDLR protein shown in Fig. 3D.
(EPS)

S6 Fig A polygenic contribution by common LDL-C risk alleles does not explain unexpected plasma LDL-C levels in ATVB *LDLR* variant carriers. For each ATVB participant genotyped by exome-chip (n = 2,433), LDL-C specific gene scores were calculated according to [30] based on the weighted sum of 20 common LDL-C raising risk alleles identified through the Global Lipid Genetics Consortium (GLGC) [48]. Carriers of *LDLR* variants identified as “disruptive-missense” in this study, but unexpectedly low LDL-C are highlighted in green, carriers of variants classified as “non-disruptive”, but high LDL-C in red (light red, “disruptive-missense” carriers with LDL-C >190mg/dl).
(EPS)

S7 Fig Principle component analysis reflects equal population structure between cases and controls. Shown are principal component analysis blots to visualize the distribution of two randomly chosen parameters (PC1, PC2; see S10 Table) between cases and controls in the ATVB cohort (n = 3,235 individuals) for (**A**) plasma LDL-C levels (with cases defined as showing LDL-C >190 mg/dl) and (**B**) MI status.
(EPS)

S1 Table Comprehensive list, allele frequencies and predicted function of LDLR missense variants discovered by exome sequencing of 3,325 participants of the ATVB study. (DOCX)

S2 Table Association of a burden of rare variants in *LDLR* with plasma LDL-C levels and MI-risk for variants classified as non-disruptive and unclear. (DOCX)

S3 Table Quantitative estimates of effect sizes (beta) based on continuous levels of LDL-C for the displayed burdens of LDLR variants. (DOCX)

S4 Table Pearson’s correlations between analyzed parameters in LDL-uptake overexpression versus complementation experiments. (DOCX)

S5 Table Impact of *LDLR* variants functionally classified in this study as disruptive-missense on free cholesterol (FC) as visualized by Filipin. (DOCX)

S6 Table A priori information from locus specific databases and the literature on putative disease relevance on all 79 LDLR missense variants functionally characterized in this study. (DOCX)

S7 Table Comparative phenotypes for all *LDLR* missense variants functionally analyzed in this study. (DOCX)

S8 Table Common variants used to determine polygenic risk scores for association with plasma LDL-C. (DOCX)

S9 Table Rare and low-frequency coding variants identified in 12 Mendelian lipid disease genes among 23 LDLR variant carriers with unexpected high or low plasma LDL-C. (DOCX)

S10 Table Distribution of MI risk factors between ATVB MI cases and controls (means). (DOCX)

S11 Table Primer sequences for site-directed mutagenesis of LDLR’-GFP. (DOCX)

S1 Spreadsheet Comprehensive results for all 79 *LDLR* missense variants functionally characterized in this study by overexpression and complementation for a role on cellular LDL-uptake. (XLSX)
