# Correction: Systematic Cell-Based Phenotyping of Missense Alleles Empowers Rare Variant Association Studies: A Case for *LDLR* and Myocardial Infarction

**DOI:** 10.1371/journal.pgen.1005060

**Published:** 2015-03-17

**Authors:** The PLOS Genetics Staff

The affiliations for the sixteenth author are incorrect. Sekar Kathiresan is affiliated not with #9 but with #3 Center of Human Genetic Research (CHGR), Massachusetts General Hospital, Boston, Massachusetts, United States of America, #4 Broad Institute of MIT and Harvard, Cambridge, Massachusetts, United States of America, #5 Cardiovascular Research Center, Massachusetts General Hospital, Boston, Massachusetts, United States of America and #6 Department of Medicine, Harvard Medical School, Boston, Massachusetts, United States of America.

Additionally the authorship contributions are incorrect. The correct contributions are equal first-author contribution for AST and HR, and an equal last-author contribution for RP, SK and HR.
