# Supplementary material for: Systematic Cell-Based Phenotyping of Missense Alleles Empowers Rare Variant Association Studies: A Case for LDLR and Myocardial Infarction
Source: PLoS Genet. 2015 Feb 3;11(2):e1004855. doi: 10.1371/journal.pgen.1004855 (PMC4409815; doi:10.1371/journal.pgen.1004855)
Supplement: S1 Table — (DOCX) [file pgen.1004855.s008.docx]

| **Table S1. Comprehensive list, allele frequencies and predicted function of LDLR missense variants discovered by exome sequencing of 3,325 participants of the ATVB study*** | | | | | | | | | | | | | | | | | | | |
| --- | --- | --- | --- | --- | --- | --- | --- | --- | --- | --- | --- | --- | --- | --- | --- | --- | --- | --- | --- |
|  | | | | | | | | | | | | | | | | | | | |
| **pos** | **ref** | **alt** | **exon** | **amino acid chg** | **rs ID** | **ClinVar**  **Accession** | **allele frequency** | **allele count** | **MI summary** | **count_MI** | **count_no MI** | **plasma LDL-C summary** | **plasma LDL-c count >190mg/dl** | **plasma LDL-c count <190mg/dl** | **PREDICTION summary** | **PolyPhen-2 score** | **SIFT score** | **MutationAssessor score** | **MutationTaster**  **probabilty** |
| 19:11200282 | G | A | 1 | **p.G20R** | rs147509697 | SCV000189524 | 0,002936631 | 19 | BOTH** | 11 | 8 | >1 LDL HIGH*** | 3 | 10 | BENIGN | 0.12 | 0,16 | 0,83 | 1 |
| 19:11210974 | G | A | 2 | **p.G48D** |  | SCV000189525 | 0,00015456 | 1 | ONLY NO MI | 0 | 1 | LDL NORMAL | 0 | 1 | DAMAGING | 1 | 0,02 | 2,55 | 1 |
| 19:11211016 | C | T | 2 | **p.T62M** | rs376207800 | SCV000189526 | 0,000772798 | 5 | BOTH | 3 | 2 | LDL NORMAL | 0 | 3 | UNCLEAR | 1 | 0,14 | 0,69 | 1 |
| 19:11213390 | C | T | 3 | **p.R81C** |  | SCV000189527 | 0,00015456 | 1 | ONLY MI | 1 | 0 | NA**** | 0 | 0 | DAMAGING | 1 | 0 | 2,36 | 1 |
| 19:11215896 | C | T | 4 | **p.P105L** |  | SCV000189528 | 0,000309119 | 2 | BOTH | 1 | 1 | LDL NORMAL | 0 | 1 | BENIGN | 0 | 0,17 | 1,90 | 1 |
| 19:11215934 | G | T | 4 | **p.D118Y** |  | SCV000189529 | 0,000309119 | 2 | ONLY MI | 2 | 0 | NA | 0 | 0 | UNCLEAR | 0,99 | 0 | 1,80 | 0,59 |
| 19:11215974 | A | G | 4 | **p.D131G** |  | SCV000189530 | 0,000154607 | 1 | ONLY MI | 1 | 0 | MEAN LDL HIGH***** | 1 | 0 | DAMAGING | 0,63 | 0 | 3,18 | 1 |
| 19:11215991 | G | A | 4 | **p.G137S** |  | SCV000189531 | 0,000154607 | 1 | ONLY NO MI | 0 | 1 | LDL NORMAL | 0 | 1 | DAMAGING | 1 | 0,01 | 2,41 | 1 |
| 19:11215992 | G | T | 4 | **p.G137V** |  | SCV000189532 | 0,000154607 | 1 | ONLY MI | 1 | 0 | MEAN LDL HIGH | 1 | 0 | DAMAGING | 1 | 0 | 3,68 | 1 |
| 19:11216112 | C | T | 4 | **p.S177L** | rs121908026 | SCV000189533 | 0,000154607 | 1 | ONLY MI | 1 | 0 | MEAN LDL HIGH | 1 | 0 | DAMAGING | 1 | 0 | 3,48 | 1 |
| 19:11216124 | C | G | 4 | **p.P181R** |  | SCV000189534 | 0,000618429 | 4 | BOTH | 3 | 1 | >1 LDL HIGH | 1 | 3 | DAMAGING | 1 | 0,29 | 2,20 | 1 |
| 19:11216127 | A | G | 4 | **p.Q182R** |  | SCV000189535 | 0,000154607 | 1 | ONLY MI | 1 | 0 | LDL NORMAL | 0 | 1 | BENIGN | 0,06 | 0,10 | 0,43 | 1 |
| 19:11216171 | T | C | 4 | **p.C197R** |  | SCV000189536 | 0,000154607 | 1 | ONLY MI | 1 | 0 | LDL NORMAL | 0 | 1 | DAMAGING | 1 | 0 | 4,61 | 1 |
| 19:11216244 | A | G | 4 | **p.D221G** | rs373822756 | SCV000189537 | 0,000927644 | 6 | ONLY MI | 6 | 0 | MEAN LDL HIGH | 2 | 2 | DAMAGING | 1 | 0 | 4,24 | 1 |
| 19:11216247 | G | A | 4 | **p.C222Y** |  | SCV000189538 | 0,000154607 | 1 | ONLY MI | 1 | 0 | NA | 0 | 0 | DAMAGING | 1 | 0 | 4,69 | 1 |
| 19:11217256-7 | GG | AC | 5 | **p.R237H** |  | SCV000189539 | 0,000309119 | 2 | ONLY NO MI | 0 | 2 | LDL NORMAL | 1 | 1 | UNCLEAR | 0,99 | 0,01 | 1,64 | 0,98 |
| 19:11217336 | A | C | 5 | **p.M264L** |  | SCV000189540 | 0,00015456 | 1 | ONLY MI | 1 | 0 | MEAN LDL HIGH | 1 | 0 | BENIGN | 0 | 0,72 | 0,28 | 0,83 |
| 19:11217352 | G | A | 5 | **p.G269D** | rs143992984 | SCV000189541 | 0,00015456 | 1 | ONLY MI | 1 | 0 | LDL NORMAL | 0 | 1 | BENIGN | 0,01 | 0,23 | 0,65 | 1 |
| 19:11218077 | G | C | 6 | **p.C276S** |  | SCV000189542 | 0,00015456 | 1 | ONLY MI | 1 | 0 | MEAN LDL HIGH | 1 | 0 | DAMAGING | 0,99 | 0,01 | 3,87 | 1 |
| 19:11218079 | G | A | 6 | **p.E277K** | rs148698650 | SCV000189543 | 0,001081917 | 7 | BOTH | 5 | 2 | LDL NORMAL | 0 | 6 | BENIGN | 0,29 | 0,64 | 1,70 | 1 |
| 19:11218096 | C | A | 6 | **p.F282L** |  | SCV000189544 | 0,00015456 | 1 | ONLY MI | 1 | 0 | LDL NORMAL | 0 | 1 | DAMAGING | 0,40 | 0 | 3,21 | 1 |
| 19:11218103 | C | T | 6 | **p.H285Y** |  | SCV000189545 | 0,00015456 | 1 | ONLY NO MI | 0 | 1 | NA | 0 | 0 | BENIGN | 0,28 | 0,06 | 1,18 | 0,74 |
| 19:11218142 | A | G | 6 | **p.M298V** |  | SCV000189546 | 0,000463679 | 3 | ONLY NO MI | 0 | 3 | LDL NORMAL | 0 | 3 | BENIGN | 0 | 0,17 | -1,01 | 1 |
| 19:11218158 | G | A | 6 | **p.R303Q** |  | SCV000189547 | 0,00015456 | 1 | ONLY MI | 1 | 0 | LDL NORMAL | 0 | 1 | BENIGN | 0,12 | 0,24 | 1,59 | 0,53 |
| 19:11218190 | G | A | 6 | **p.G314R** | rs72658858 | SCV000189548 | 0,00015456 | 1 | ONLY NO MI | 0 | 1 | NA | 0 | 0 | BENIGN | 0,11 | 0,15 | 1,87 | 1 |
| 19:11221334 | A | G | 7 | **p.N316S** |  | SCV000189549 | 0,000463679 | 3 | BOTH | 2 | 1 | LDL NORMAL | 0 | 2 | DAMAGING | 0,81 | 0,01 | 3,07 | 1 |
| 19:11221375 | A | C | 7 | **p.N330H** |  | SCV000189550 | 0,000309119 | 2 | BOTH | 1 | 1 | NA | 0 | 0 | BENIGN | 0,56 | 0,20 | -0,09 | 0,78 |
| 19:11221390 | G | A | 7 | **p.G335S** |  | SCV000189551 | 0,000309119 | 2 | BOTH | 1 | 1 | LDL NORMAL | 0 | 2 | DAMAGING | 1 | 0,04 | 0,75 | 1 |
| 19:11221414 | G | A | 7 | **p.G343S** |  | SCV000189552 | 0,00015456 | 1 | ONLY NO MI | 0 | 1 | NA | 0 | 0 | DAMAGING | 1 | 0 | 3,75 | 1 |
| 19:11221444 | G | A | 7 | **p.E353K** | rs370471092 | SCV000189553 | 0,00015456 | 1 | ONLY MI | 1 | 0 | LDL NORMAL | 0 | 1 | BENIGN | 0,36 | 0,17 | 1,74 | 1 |
| 19:11222234 | G | A | 8 | **p.V369M** |  | SCV000189554 | 0,00015456 | 1 | ONLY NO MI | 0 | 1 | LDL NORMAL | 0 | 1 | DAMAGING | 0,99 | 0 | 1,54 | 1 |
| 19:11222262 | A | C | 8 | **p.Q378P** |  | SCV000189555 | 0,00015456 | 1 | ONLY MI | 1 | 0 | LDL NORMAL | 0 | 1 | UNCLEAR | 0,16 | 0,01 | 1,85 | 0,99 |
| 19:11223962 | G | A | 8 | **p.A399T** |  | SCV000189556 | 0,000309119 | 2 | BOTH | 1 | 1 | MEAN LDL HIGH | 1 | 1 | DAMAGING | 0,99 | 0,01 | 2,01 | 1 |
| 19:11224013 | C | T | 9 | **p.R416W** |  | SCV000189557 | 0,00015456 | 1 | ONLY MI | 1 | 0 | MEAN LDL HIGH | 1 | 0 | DAMAGING | 1 | 0 | 2,37 | 1 |
| 19:11224061 | C | G | 9 | **p.L432V** |  | SCV000189558 | 0,000309119 | 2 | ONLY MI | 2 | 0 | MEAN LDL HIGH | 1 | 1 | DAMAGING | 0,95 | 0 | 2,38 | 1 |
| 19:11224103 | C | G | 9 | **p.L446V** |  | SCV000189559 | 0,00015456 | 1 | ONLY MI | 1 | 0 | MEAN LDL HIGH | 1 | 0 | BENIGN | 0,01 | 0,41 | 1,08 | 1 |
| 19:11224233 | G | T | 10 | **p.G461C** |  | SCV000189560 | 0,00015456 | 1 | ONLY MI | 1 | 0 | NA | 0 | 0 | BENIGN | 0,42 | 0,04 | 1,85 | 1 |
| 19:11224245 | T | A | 10 | **p.Y465N** |  | SCV000189561 | 0,000309119 | 2 | ONLY MI | 2 | 0 | MEAN LDL HIGH | 1 | 1 | BENIGN | 0,02 | 0,10 | 1,53 | 0,64 |
| 19:11224254 | G | A | 10 | **p.V468I** | rs5932 | SCV000189562 | 0,000309119 | 2 | ONLY MI | 2 | 0 | LDL NORMAL | 0 | 2 | BENIGN | 0,02 | 0,23 | 1,04 | 1 |
| 19:11224266 | G | T | 10 | **p.D472Y** |  | SCV000189563 | 0,000772798 | 5 | ONLY MI | 5 | 0 | MEAN LDL HIGH | 1 | 0 | DAMAGING | 0,78 | 0,01 | 3,11 | 1 |
| 19:11224326 | G | A | 10 | **p.D492N** | rs373646964 | SCV000189564 | 0,00015456 | 1 | ONLY MI | 1 | 0 | LDL NORMAL | 0 | 1 | DAMAGING | 1 | 0 | 2,88 | 1 |
| 19:11224362 | A | G | 10 | **p.K504E** |  | SCV000189565 | 0,000309119 | 2 | ONLY MI | 2 | 0 | MEAN LDL HIGH | 1 | 0 | BENIGN | 0 | 0,29 | 0,30 | 0,85 |
| 19:11224399 | G | A | 10 | **p.G516D** |  | SCV000189566 | 0,00015456 | 1 | ONLY NO MI | 0 | 1 | LDL NORMAL | 0 | 1 | BENIGN | 0,02 | 0,16 | 1,59 | 1 |
| 19:11224419 | G | A | 10 | **p.V523M** | rs28942080 | SCV000189567 | 0,00015456 | 1 | ONLY MI | 1 | 0 | NA | 0 | 0 | DAMAGING | 1 | 0 | 3,61 | 1 |
| 19:11224422 | G | A | 10 | **p.V524M** |  | SCV000189568 | 0,00015456 | 1 | ONLY MI | 1 | 0 | LDL NORMAL | 0 | 1 | DAMAGING | 0,97 | 0 | 3,52 | 1 |
| 19:11224428 | C | T | 10 | **p.P526S** |  | SCV000189569 | 0,00015456 | 1 | ONLY NO MI | 0 | 1 | MEAN LDL HIGH | 1 | 0 | DAMAGING | 1 | 0 | 3,21 | 1 |
| 19:11224432 | T | C | 10 | **p.V527A** |  | SCV000189570 | 0,00015456 | 1 | ONLY NO MI | 0 | 1 | NA | 0 | 0 | BENIGN | 0 | 0,15 | 0,16 | 1 |
| 19:11224437 | G | C | 10 | **p.G529R** |  | SCV000189571 | 0,000309119 | 2 | ONLY MI | 2 | 0 | LDL NORMAL | 0 | 2 | DAMAGING | 1 | 0,03 | 2,30 | 1 |
| 19:11226829 | G | A | 11 | **p.G549D** | rs28941776 | SCV000189572 | 0,000618238 | 4 | ONLY MI | 4 | 0 | MEAN LDL HIGH | 3 | 0 | DAMAGING | 1 | 0 | 4,09 | 1 |
| 19:11227549 | C | T | 12 | **p.R574C** | rs185098634 | SCV000189573 | 0,00015456 | 1 | ONLY MI | 1 | 0 | MEAN LDL HIGH | 1 | 0 | DAMAGING | 1 | 0 | 3,69 | 1 |
| 19:11227576 | C | G | 12 | **p.H583D** |  | SCV000189574 | 0,000154847 | 1 | ONLY MI | 1 | 0 | MEAN LDL HIGH | 1 | 0 | DAMAGING | 1 | 0,01 | 0,67 | 1 |
| 19:11227594 | G | C | 12 | **p.D589H** |  | SCV000189575 | 0,000154943 | 1 | ONLY MI | 1 | 0 | LDL NORMAL | 0 | 1 | DAMAGING | 1 | 0 | 2,26 | 1 |
| 19:11227604 | G | A | 12 | **p.G592E** | rs137929307 | SCV000189576 | 0,000464828 | 3 | BOTH | 2 | 1 | MEAN LDL HIGH | 2 | 1 | DAMAGING | 0,88 | 0 | 3,42 | 1 |
| 19:11227613 | G | A | 12 | **p.R595Q** | rs201102492 | SCV000189577 | 0,000309885 | 2 | ONLY MI | 2 | 0 | >1 LDL HIGH | 1 | 1 | DAMAGING | 1 | 0 | 3,34 | 1 |
| 19:11227645 | G | T | 12 | **p.A606S** | rs72658865 | SCV000189578 | 0,00015528 | 1 | ONLY MI | 1 | 0 | LDL NORMAL | 0 | 1 | BENIGN | 0,07 | 0,32 | 0,62 | 1 |
| 19:11230798 | G | A | 13 | **p.E626K** | rs139791325 | SCV000189579 | 0,00015456 | 1 | ONLY MI | 1 | 0 | LDL NORMAL | 0 | 1 | BENIGN | 0,64 | 0,21 | 1,17 | 0,87 |
| 19:11230873 | G | A | 13 | **p.D651N** |  | SCV000189580 | 0,00015456 | 1 | ONLY MI | 1 | 0 | NA | 0 | 0 | DAMAGING | 0,19 | 0,02 | 3,11 | 1 |
| 19:11230876 | A | G | 13 | **p.M652V** |  | SCV000189581 | 0,00015456 | 1 | ONLY NO MI | 0 | 1 | NA | 0 | 0 | BENIGN | 0 | 0,10 | -1,24 | 0,98 |
| 19:11231112 | C | T | 14 | **p.P685L** | rs28942084 | SCV000189582 | 0,000309119 | 2 | ONLY MI | 2 | 0 | MEAN LDL HIGH | 2 | 0 | DAMAGING | 1 | 0 | 3,27 | 1 |
| 19:11231159 | G | A | 14 | **p.G701S** | rs368838866 | SCV000189583 | 0,000309119 | 2 | BOTH | 1 | 1 | LDL NORMAL | 0 | 2 | DAMAGING | 0,99 | 0,03 | 2,76 | 0,95 |
| 19:11231174 | A | G | 14 | **p.R706G** |  | SCV000189584 | 0,00015456 | 1 | ONLY NO MI | 0 | 1 | LDL NORMAL | 0 | 1 | BENIGN | 0 | 0,15 | 0,19 | 1 |
| 19:11231184 | G | A | 14 | **p.R709K** |  | SCV000189585 | 0,000309119 | 2 | ONLY MI | 2 | 0 | LDL NORMAL | 0 | 1 | UNCLEAR | 0,98 | 0,53 | 0,67 | 1 |
| 19:11233886 | C | T | 15 | **p.T726I** | rs45508991 | SCV000189586 | 0,00618238 | 40 | BOTH | 24 | 16 | >1 LDL HIGH | 3 | 24 | BENIGN | 0,02 | 0,51 | 2,41 | 1 |
| 19:11233940 | G | A | 15 | **p.R744Q** | rs137853963 | SCV000189587 | 0,000772798 | 5 | BOTH | 2 | 3 | LDL NORMAL | 0 | 4 | BENIGN | 0 | 0,59 | 1,15 | 1 |
| 19:11233951 | G | A | 15 | **p.D748N** | rs150104358 | SCV000189588 | 0,00015456 | 1 | ONLY MI | 1 | 0 | MEAN LDL HIGH | 1 | 0 | BENIGN | 0 | 0,41 | -0,35 | 1 |
| 19:11233991 | C | T | 15 | **p.T761M** | rs138477254 | SCV000189589 | 0,00015456 | 1 | ONLY MI | 1 | 0 | LDL NORMAL | 0 | 1 | DAMAGING | 1 | 0,05 | 2,11 | 0,94 |
| 19:11238728 | A | G | 16 | **p.S786G** |  | SCV000189590 | 0,00015456 | 1 | ONLY NO MI | 0 | 1 | NA | 0 | 0 | BENIGN | 0 | 1 | 0,42 | 1 |
| 19:11240197 | G | A | 17 | **p.V800I** | rs200243555 | SCV000189591 | 0,00015456 | 1 | ONLY NO MI | 0 | 1 | LDL NORMAL | 0 | 1 | BENIGN | 0 | 0,27 | 0,62 | 1 |
| 19:11240278 | G | A | 17 | **p.V827I** | rs137853964 | SCV000189592 | 0,00015456 | 1 | ONLY NO MI | 0 | 1 | NA | 0 | 0 | DAMAGING | 1 | 0,06 | 2,14 | 1 |
| 19:11241984 | G | A | 18 | **p.V859M** | rs202049029 | NA | 0,00015456 | 1 | ONLY MI | 1 | 0 | MEAN LDL HIGH | 1 | 0 | BENIGN | 0,03 | 0,06 | 0,90 | 1 |
| * 70 LDLR missense variants (non-synonymous coding) identified among participants of the ATVB study cohort were classified according to (i) occurence in MI versus MI-free ("no MI") individuals, (ii) occurence in individuals with highly elevated plasma LDL-C (>190mg/dl) or LDL-C defined as "normal" (<190mg/dl) and (iii) functional impact as predicted by the overlap of four bioinformatic prediction tools, amino acid numbers refer to LDLR RefSeq transcript NM_000527.4 (ENST00000558518; 860aa)  ** "BOTH", variant is present in MI cases as well as MI-free controls ("no MI"); ***">1 LDL HIGH", at least one carrier showed LDL-C>190mg/dl; **** "NA", not available; *****"MEAN LDL HIGH", the mean of all individuals with this variant was >190mg/dl  PREDICTION summary", reflecting the overlap of prediction tools PolyPhen-2, SIFT, MutationAssessor and MutationTaster (see Methods) | | | | | | | | | | | | | | | | | | | |
