# Supplementary material for: Systematic Cell-Based Phenotyping of Missense Alleles Empowers Rare Variant Association Studies: A Case for LDLR and Myocardial Infarction
Source: PLoS Genet. 2015 Feb 3;11(2):e1004855. doi: 10.1371/journal.pgen.1004855 (PMC4409815; doi:10.1371/journal.pgen.1004855)
Supplement: S2 Table — (DOCX) [file pgen.1004855.s009.docx]

| **Table S2. Association of a burden of rare variants in *LDLR* with plasma LDL-C levels and MI-risk for variants classified as non-disruptive and unclear.** | | | | | | | | | |
| --- | --- | --- | --- | --- | --- | --- | --- | --- | --- |
|  | | | | | | | | | |
| **pheno-**  **type** | **variants analyzed** | **variant count** | **allele count** | **allele freq.** | **LDL-C >190mg/dl**  **(n=251)** | **LDL-C <190mg/dl**  **(n=1,901)** | **P-value** | **OR** | **95% CI** |
| **plasma**  **LDL-C** | non-disruptive | 46 | 99 | 0.046 | 19 | 80 | 0.023 | 1.8 | 1.0-3.1 |
|  | unclear | 10 | 10 | 0.005 | 2 | 8 | 0.330 | 1.9 | 0.2-9.6 |
|  | non-disruptive + unclear | 56 | 109 | 0.051 | 21 | 88 | 0.038 | 1.8 | 1.0-3.1 |
| **pheno-**  **type** | **variants analyzed** | **variant count** | **allele count** | **allele freq.** | **MI case**  **(n=1,716)** | **MI control**  **(n=1,519)** | **P-value** | **OR** | **95% CI** |
| **MI** | non-disruptive | 46 | 136 | 0.042 | 82 | 54 | 0.035 | 1.3 | 0.9-1.9 |
|  | unclear | 10 | 12 | 0.004 | 10 | 2 | 0.043 | 4.4 | 0.9-41.6 |
|  | non-disruptive + unclear | 56 | 148 | 0.046 | 92 | 56 | 0.114 | 1.5 | 1.0-2.1 |
