# Supplementary material for: Systematic Cell-Based Phenotyping of Missense Alleles Empowers Rare Variant Association Studies: A Case for LDLR and Myocardial Infarction
Source: PLoS Genet. 2015 Feb 3;11(2):e1004855. doi: 10.1371/journal.pgen.1004855 (PMC4409815; doi:10.1371/journal.pgen.1004855)
Supplement: S3 Table — (DOCX) [file pgen.1004855.s010.docx]

| **Table S3. Quantitative estimates of effect sizes (beta) based on continuous levels of LDL-C for the displayed burdens of LDLR variants.** | | | | | | | |
| --- | --- | --- | --- | --- | --- | --- | --- |
|  | | | | | | | |
| **variants analyzed** | **variant count** | **allele count** | **allele**  **freq.** | **P-value** | **Beta** | **95% CI** |  |
| clear LoF | 11 | 16 | 0.007 | 9.9x10^-36^ | 142.6 | 120.6-164.6 |  |
| all missense | 55 | 127 | 0.059 | 2.9x10^-9^ | 25.4 | 17.0-33.7 |  |
| all missense + LoF | 66 | 143 | 0.066 | 2.2x10^-23^ | 39.9 | 32.1-47.7 |  |
| disruptive-missense | 13 | 20 | 0.009 | 2.9x10^-16^ | 84.4 | 64.4-104.5 |  |
| disruptive missense + LoF | 24 | 36 | 0.017 | 1.6x10^-48^ | 111.3 | 96.7-125.8 |  |
