# Supplementary material for: Systematic Cell-Based Phenotyping of Missense Alleles Empowers Rare Variant Association Studies: A Case for LDLR and Myocardial Infarction
Source: PLoS Genet. 2015 Feb 3;11(2):e1004855. doi: 10.1371/journal.pgen.1004855 (PMC4409815; doi:10.1371/journal.pgen.1004855)
Supplement: S4 Table — (DOCX) [file pgen.1004855.s011.docx]

| **Table S4. Pearson’s correlations between analyzed parameters in LDL-uptake overexpression versus complementation experiments.*** | | | | | | | | | |
| --- | --- | --- | --- | --- | --- | --- | --- | --- | --- |
|  | | | | | | | | | |
|  |  | **overexpression** | | | | **complementation** | | | |
|  |  | **total LDL** | **LDL conc** | **seg. num.** | **seg. area** | **total LDL** | **LDL conc** | **seg. numb** | **seg. area** |
| **overexpression** | **total LDL** | 1,00 | 0,92 | 0,77 | 0,97 | 0,76 | 0,64 | 0,36 | 0,71 |
|  | **LDL conc** |  | 1,00 | 0,70 | 0,83 | 0,79 | 0,59 | 0,42 | 0,75 |
|  | **seg. num.** |  |  | 1,00 | 0,84 | 0,62 | 0,57 | 0,29 | 0,54 |
|  | **seg. area** |  |  |  | 1,00 | 0,73 | 0,67 | 0,30 | 0,67 |
| **complementation** | **total LDL** |  |  |  |  | 1,00 | 0,86 | 0,37 | 0,85 |
|  | **LDL conc** |  |  |  |  |  | 1,00 | 0,03 | 0,61 |
|  | **seg. num.** |  |  |  |  |  |  | 1,00 | 0,70 |
|  | **seg. area** |  |  |  |  |  |  |  | 1,00 |
| * Pearson's correlation among the four DiI-LDL parameters analyzed per cell and both experimental conditions.  Calculations are based on the comprehensive set of mutated LDLR'-GFP cDNAs analyzed in this study (see Table S2). | | | | | | | | | |
