# Supplementary material for: Systematic Cell-Based Phenotyping of Missense Alleles Empowers Rare Variant Association Studies: A Case for LDLR and Myocardial Infarction
Source: PLoS Genet. 2015 Feb 3;11(2):e1004855. doi: 10.1371/journal.pgen.1004855 (PMC4409815; doi:10.1371/journal.pgen.1004855)
Supplement: S5 Table — (DOCX) [file pgen.1004855.s012.docx]

| **Table S5. Impact of *LDLR* variants functionally classified in this study as disruptive-missense on free cholesterol (FC) as visualized by Filipin.*** | | | | | | | | |
| --- | --- | --- | --- | --- | --- | --- | --- | --- |
|  | | | | | | | | |
|  |  | | | **overexpression Total Filipin** | | | | |
| **source** | **exon** | **amino acid change** | **n (exp.)** | **Mean** | **SD** | **SEM** | **T-Test LDLR** | **T-Test GFP** |
| NA | NA | **GFP control** | 3 | 1,15 | 0,12 | 0,07 | 3,09E-03 | NA |
| NA | NA | **WT** | 4 | 3,56 | 0,75 | 0,38 | NA | 3,09E-03 |
| exome chip | 3 | **E101K** | 4 | 1,81 | 0,79 | 0,40 | 1,87E-02 | 2,26E-01 |
| ATVB | 4 | **D131G** | 4 | 2,23 | 0,06 | 0,03 | 1,27E-02 | 1,89E-05 |
| ATVB | 4 | **G137V** | 4 | 1,10 | 0,05 | 0,03 | 6,23E-04 | 4,23E-01 |
| exome chip | 4 | **D168N** | 4 | 1,98 | 0,57 | 0,29 | 1,58E-02 | 6,06E-02 |
| ATVB | 4 | **S177L** | 4 | 1,75 | 0,24 | 0,12 | 3,77E-03 | 1,24E-02 |
| ATVB | 4 | **C197R** | 3 | 1,38 | 0,12 | 0,07 | 4,69E-03 | 9,10E-02 |
| ATVB | 4 | **D221G** | 4 | 1,45 | 0,25 | 0,13 | 1,84E-03 | 1,21E-01 |
| ATVB | 4 | **C222Y** | 4 | 1,03 | 0,38 | 0,19 | 9,64E-04 | 6,04E-01 |
| ATVB | 6 | **C276S** | 4 | 2,11 | 0,83 | 0,42 | 4,18E-02 | 1,11E-01 |
| ATVB | 6 | **F282L** | 4 | 1,85 | 0,44 | 0,22 | 7,85E-03 | 4,99E-02 |
| ATVB | 7 | **N316S** | 1 | 1,58 | NA | NA | NA | NA |
| ATVB | 10 | **D472Y** | 4 | 3,10 | 0,91 | 0,45 | 4,69E-01 | 1,54E-02 |
| ATVB | 10 | **P526S** | 3 | 1,23 | 0,27 | 0,15 | 4,05E-03 | 6,80E-01 |
| ATVB | 11 | **G549D** | 4 | 1,23 | 0,30 | 0,15 | 1,22E-03 | 7,16E-01 |
| ATVB | 12 | **H583D** | 3 | 1,56 | 0,48 | 0,28 | 1,05E-02 | 2,31E-01 |
| ATVB, exome chip | 14 | **P685L** | 2 | 1,06 | 0,51 | 0,36 | 1,46E-02 | 7,57E-01 |
| FH control | 17 | **Y828C** | 4 | 1,80 | 0,54 | 0,27 | 9,04E-03 | 1,03E-01 |
| * for definition of parameters, see Methods and Blattmann et al., 2013 | | | | | | | | |
