# Supplementary material for: Systematic Cell-Based Phenotyping of Missense Alleles Empowers Rare Variant Association Studies: A Case for LDLR and Myocardial Infarction
Source: PLoS Genet. 2015 Feb 3;11(2):e1004855. doi: 10.1371/journal.pgen.1004855 (PMC4409815; doi:10.1371/journal.pgen.1004855)
Supplement: S6 Table — (DOCX) [file pgen.1004855.s013.docx]

| **Table S6. A priori information from locus specific databases and the literature on putative disease relevance on all 79 LDLR missense variants functionally characterized in this study.** | | | | | | |
| --- | --- | --- | --- | --- | --- | --- |
|  | | | | | | |
|  |  | **UCL LOVD** | **UMD** | **NCBI ClinVar** | **HGMD** | **Source articles**  **(see Supplementary References)** |
| **variant** | **functional class** | **reported impact on LDLR activity** | **reported phenotypes** | | |  |
| **G20R** | non-disruptive |  |  |  | + | 4, 9, 29, 37, 67, 94, 109 |
| **G48D** | non-disruptive |  |  |  |  |  |
| **T62M** | non-disruptive |  |  |  | + | 6, 9, 29, 37, 69 |
| **R81C** | unclear |  |  |  | + | 9, 36, 74, 83 |
| **E101K** | disruptive missense | 15-30% in homozygotes | + |  | + | 40, 41, 75, 87, 107 |
| **P105L** | non-disruptive |  |  |  |  |  |
| **D118Y** | unclear | 30% in heterozygotes |  |  | + | 8,9 |
| **D131G** | disruptive missense |  |  |  |  |  |
| **G137S** | non-disruptive |  |  |  | + | 22 |
| **G137V** | disruptive missense |  |  |  |  |  |
| **D168N** | disruptive missense |  |  |  | + | 24, 67, 68, 85 |
| **S177L** | disruptive missense | <2% in homozygotes | + | + | + | 18, 44, 50, 51, 91 |
| **P181R** | non-disruptive |  |  |  | + | 9, 19, 37, 39, 65, 66 |
| **Q182R** | non-disruptive |  |  |  |  |  |
| **C197R** | disruptive missense |  |  |  | + | 9, 106 |
| **G219D** | non-disruptive | little or no effect |  |  | + | 68, 69, 81 |
| **D221G** | disruptive missense | <8% in homozygotes | + |  | + | 7, 9, 42, 48, 50, 70 |
| **C222Y** | disruptive missense |  | + |  | + | 7, 9, 62 |
| **R237H** | non-disruptive |  |  |  |  |  |
| **R253W** | non-disruptive |  |  |  | + | 36, 53, 54, 58, 82 |
| **M264L** | non-disruptive |  |  |  |  |  |
| **D266E** | unclear | 15-30% in homozygotes | + |  | + | 9, 12, 36, 50, 90, 108 |
| **G269D** | non-disruptive |  |  |  | + | 9, 35, 80 |
| **C276S** | disruptive missense |  |  |  |  |  |
| **E277K** | non-disruptive | little or no effect |  |  | + | 9, 17, 32, 33, 58, 69, 84, 94, 99, 104 |
| **F282L** | disruptive missense |  |  |  | + | 30 |
| **H285Y** | non-disruptive |  |  |  |  |  |
| **M298V** | non-disruptive |  |  |  |  |  |
| **R303Q** | unclear |  |  |  |  |  |
| **G314R** | non-disruptive |  |  |  | + | 6, 109 |
| **N316S** | disruptive missense |  |  |  | + | 20 |
| **G324S** | non-disruptive |  |  |  | + | 1, 4, 5, 6, 36 |
| **N330H** | non-disruptive |  |  |  |  |  |
| **G335S** | non-disruptive | 30-40% in heterozygotes | + |  | + | 9, 50, 67, 105 |
| **D342N** | non-disruptive |  |  |  | + | 24, 29, 36, 69 |
| **G343S** | unclear | 15-30% when heterozygous with FH Baltimore-1 | + |  | + | 9, 36, 50, 53, 80, 86, 100 |
| **E353K** | unclear |  |  |  | + | 22, 29, 36, 53 |
| **V369M** | non-disruptive |  |  |  |  |  |
| **Q378P** | unclear |  |  |  | + | 9, 14, 80 |
| **A391T** | non-disruptive | little or no effect |  |  | + | 12, 34, 38, 64, 103, 105, 111 |
| **A399T** | non-disruptive |  |  |  | + | 9, 28, 37, 80 |
| **R416W** | non-disruptive |  |  |  | + | 9, 24, 36 |
| **L432V** | non-disruptive |  |  |  | + | 31 |
| **L446V** | non-disruptive |  |  |  |  |  |
| **G461C** | non-disruptive |  |  |  | + | 9, 16, 79 |
| **Y465N** | non-disruptive |  |  |  |  |  |
| **V468I** | non-disruptive |  |  |  |  |  |
| **D472Y** | disruptive missense |  |  |  | + | 9, 15 |
| **D492N** | non-disruptive |  |  |  | + | 4, 9, 22, 76, 98 |
| **K504E** | non-disruptive |  |  |  | + | 3 |
| **G516D** | non-disruptive |  |  |  |  |  |
| **V523M** | non-disruptive | 30% when compound-heterozygous with another unclassified FH mutation | + | + | + | 4, 8, 9, 52, 73, 80, 88 |
| **V524M** | non-disruptive |  |  |  | + | 9, 47 |
| **P526S** | disruptive missense | 5-15% in heterozygotes | + |  | + | 24, 50, 82 |
| **V527A** | non-disruptive |  |  |  |  |  |
| **G529R** | non-disruptive |  |  |  |  |  |
| **G549D** | disruptive missense | <2% in homozygotes | + | + | + | 9, 24, 36, 52, 80, 88, 101 |
| **R574C** | non-disruptive |  |  |  | + | 9, 22, 82 |
| **H583D** | disruptive missense |  |  |  | + | 9 |
| **D589H** | non-disruptive |  |  |  |  |  |
| **G592E** | non-disruptive | <5% when compound-heterozygous with another unclassified FH mutation | + |  | + | 9, 11, 44, 50, 53, 80, 88, 111 |
| **R595Q** | non-disruptive |  |  |  | + | 37, 59, 78, 102 |
| **A606S** | non-disruptive |  |  |  | + | 25, 29, 37, 69, 95, 96, |
| **E626K** | unclear |  |  |  | + | 37 |
| **D651N** | non-disruptive |  |  |  | + | 80 |
| **M652V** | non-disruptive |  |  |  | + |  |
| **P685L** | disruptive missense | 15-30% in homozygotes | + | + | + | 9, 27, 53, 60, 61, 76, 77, 89, 92, 93, 97 |
| **G701S** | unclear |  |  |  | + | 37, 53 |
| **R706G** | non-disruptive |  |  |  |  |  |
| **R709K** | non-disruptive |  |  |  |  |  |
| **T726I** | non-disruptive | little or no effect ;15-30% when compound-heterozygous with p.E240K |  | + | + | 2, 5, 6, 9, 11, 13, 21, 24, 26, 31, 43, 45, 46, 49, 50, 53, 54, 56, 57, 69, 72, 73, 75, 80, 99, 103 |
| **R744Q** | non-disruptive | little or no effect |  |  | + | 4, 10, 22, 37, 53, 54, 69, 95, 96, 111 |
| **D748N** | unclear |  |  |  |  |  |
| **T761M** | non-disruptive |  |  |  | + | 12, 37, 53, 54 |
| **S786G** | non-disruptive |  |  |  |  |  |
| **V800I** | non-disruptive |  |  |  |  |  |
| **R814Q** | non-disruptive |  |  |  | + | 5, 29, 37, 55, 100 |
| **V827I** | non-disruptive | 15-30% when compound-heterozygous with FH Lithuania | + | + | + | 9, 10, 22, 29, 43, 50, 53, 54, 63, 67, 71, 110 |
| **Y828C** | unclear | <2% when compound-heterozygous with FH Potenz | + | + | + | 9, 23, 86 |
| +, listed as potentially causative for hypercholesterolemia, familial hypercholesterolemia (FH), stroke, or coronary artery disease | | | | | | |

**SUPPLEMENTAL REFERENCES (TO TABLE S6)**

1. Abecasis GR, Altshuler D, Auton A, Brooks LD, Durbin RM, et al. (2010) A map of human genome variation from population-scale sequencing. Nature 467: 1061-1073.

2. Abifadel M, Rabes JP, Jambart S, Halaby G, Gannage-Yared MH, et al. (2009) The molecular basis of familial hypercholesterolemia in Lebanon: spectrum of LDLR mutations and role of PCSK9 as a modifier gene. Hum Mutat 30: E682-691.

3. Alonso R, Defesche JC, Tejedor D, Castillo S, Stef M, et al. (2009) Genetic diagnosis of familial hypercholesterolemia using a DNA-array based platform. Clin Biochem 42: 899-903.

4. Amsellem S, Briffaut D, Carrie A, Rabes JP, Girardet JP, et al. (2002) Intronic mutations outside of Alu-repeat-rich domains of the LDL receptor gene are a cause of familial hypercholesterolemia. Hum Genet 111: 501-510.

5. Arca M, Jokinen E (1998) Low density lipoprotein receptor mutations in a selected population of individuals with moderate hypercholesterolemia. Atherosclerosis 136: 187-194.

6. Berg JS, Adams M, Nassar N, Bizon C, Lee K, et al. (2013) An informatics approach to analyzing the incidentalome. Genet Med 15: 36-44.

7. Bertolini S, Cantafora A, Averna M, Cortese C, Motti C, et al. (2000) Clinical expression of familial hypercholesterolemia in clusters of mutations of the LDL receptor gene that cause a receptor-defective or receptor-negative phenotype. Arterioscler Thromb Vasc Biol 20: E41-52.

8. Bertolini S, Cassanelli S, Garuti R, Ghisellini M, Simone ML, et al. (1999) Analysis of LDL Receptor Gene Mutations in Italian Patients With Homozygous Familial Hypercholesterolemia. Arterioscler Thromb Vasc Biol 19: 408-418.

9. Bertolini S, Pisciotta L, Rabacchi C, Cefalu AB, Noto D, et al. (2013) Spectrum of mutations and phenotypic expression in patients with autosomal dominant hypercholesterolemia identified in Italy. Atherosclerosis 227: 342-348.

10. Biesecker LG, Mullikin JC, Facio FM, Turner C, Cherukuri PF, et al. (2009) The ClinSeq Project: piloting large-scale genome sequencing for research in genomic medicine. Genome Res 19: 1665-1674.

11. Bochmann H, Geisel J, Herrmann W, Purcz T, Reuter W, et al. (2001) Eight novel LDL receptor gene mutations among patients under LDL apheresis in Dresden and Leipzig. Hum Mutat 17: 76-77.

12. Brusgaard K, Jordan P, Hansen H, Hansen AB, Horder M (2006) Molecular genetic analysis of 1053 Danish individuals with clinical signs of familial hypercholesterolemia. Clin Genet 69: 277-283.

13. Bunn CF, Lintott CJ, Scott RS, George PM (2002) Comparison of SSCP and DHPLC for the detection of LDLR mutations in a New Zealand cohort. Hum Mutat 19: 311.

14. Callis M, Jansen S, Thiart R, de Villiers JN, Raal FJ, et al. (1998) Mutation analysis in familial hypercholesterolemia patients of different ancestries: identification of three novel LDLR gene mutations. Mol Cell Probes 12: 149-152.

15. Campagna F, Martino F, Bifolco M, Montali A, Martino E, et al. (2008) Detection of familial hypercholesterolemia in a cohort of children with hypercholesterolemia: results of a family and DNA-based screening. Atherosclerosis 196: 356-364.

16. Cefalu AB, Barraco G, Noto D, Valenti V, Barbagallo CM, et al. (2006) Six novel mutations of the LDL receptor gene in FH kindred of Sicilian and Paraguayan descent. Int J Mol Med 17: 539-546.

17. Cenarro A, Jensen HK, Casao E, Civeira F, Gonzalez-Bonillo J, et al. (1998) Identification of recurrent and novel mutations in the LDL receptor gene in Spanish patients with familial hypercholesterolemia. Mutations in brief no. 135. Online. Hum Mutat 11: 413.

18. Cenarro A, Jensen HK, Civeira F, Casao E, Ferrando J, et al. (1996) Two novel mutations in the LDL receptor gene: common causes of familial hypercholesterolemia in a Spanish population. Clin Genet 49: 180-185.

19. Chaves FJ, Real JT, Garcia-Garcia AB, Civera M, Armengod ME, et al. (2001) Genetic diagnosis of familial hypercholesterolemia in a South European outbreed population: influence of low-density lipoprotein (LDL) receptor gene mutations on treatment response to simvastatin in total, LDL, and high-density lipoprotein cholesterol. J Clin Endocrinol Metab 86: 4926-4932.

20. Chiou KR, Charng MJ (2010) Detection of mutations and large rearrangements of the low-density lipoprotein receptor gene in Taiwanese patients with familial hypercholesterolemia. Am J Cardiol 105: 1752-1758.

21. Civeira F, Jarauta E, Cenarro A, Garcia-Otin AL, Tejedor D, et al (2008) Frequency of low-density lipoprotein receptor gene mutations in patients with a clinical diagnosis of familial combined hyperlipidemia in a clinical setting. J Am Coll Cardiol 52: 1546-1553.

22. Damgaard D, Larsen ML, Nissen PH, Jensen JM, Jensen HK, et al. (2005) The relationship of molecular genetic to clinical diagnosis of familial hypercholesterolemia in a Danish population. Atherosclerosis 180: 155-160.

23. Davis CG, Lehrman MA, Russell DW, Anderson RGW, Brown MS, et al. (1986) The J. D. mutation in familial hypercholesterolemia: Amino acid substitution in cytoplasmic domain impedes internalization of LDL receptors. Cell 45: 15-24.

24. Day IN, Whittall RA, O'Dell SD, Haddad L, Bolla MK, et al. (1997) Spectrum of LDL receptor gene mutations in heterozygous familial hypercholesterolemia. Hum Mutat 10: 116-127.

25. Dedoussis GV, Genschel J, Bochow B, Pitsavos C, Skoumas J, et al. (2004) Molecular characterization of familial hypercholesterolemia in German and Greek patients. Hum Mutat 23: 285-286.

26. Defesche JC, Schuurman EJ, Klaaijsen LN, Khoo KL, Wiegman A, et al. (2008) Silent exonic mutations in the low-density lipoprotein receptor gene that cause familial hypercholesterolemia by affecting mRNA splicing. Clin Genet 73: 573-578.

27. Defesche JC, van de Ree MA, Kastelein JJ, van Diermen DE, Janssens NW, et al. (1992) Detection of the Pro664-Leu mutation in the low-density lipoprotein receptor and its relation to lipoprotein(a) levels in patients with familial hypercholesterolemia of Dutch ancestry from The Netherlands and Canada. Clin Genet 42: 273-280.

28. Deiana L, Garuti R, Pes GM, Carru C, Errigo A, et al. (2000) Influence of beta(0)-thalassemia on the phenotypic expression of heterozygous familial hypercholesterolemia: a study of patients with familial hypercholesterolemia from Sardinia. Arterioscler Thromb Vasc Biol 20: 236-243.

29. Dorschner MO, Amendola LM, Turner EH, Robertson PD, Shirts BH, et al. (2013) Actionable, Pathogenic Incidental Findings in 1,000 Participants Exomes. Am J Hum Genet 93: 631-640.

30. Duskova L, Kopeckova L, Jansova E, Tichy L, Freiberger T, et al. (2011) An APEX-based genotyping microarray for the screening of 168 mutations associated with familial hypercholesterolemia. Atherosclerosis 216: 139-145.

31. Ebhardt M, Schmidt H, Doerk T, Tietge U, Haas R, et al. (1999) Mutation analysis in 46 German families with familial hypercholesterolemia: identification of 8 new mutations. Mutations in brief no. 226. Online. Hum Mutat 13: 257.

32. Ekstrom U, Abrahamson M, Sveger T, Lombardi P, Nilsson-Ehle P (1995) An efficient screening procedure detecting six novel mutations in the LDL receptor gene in Swedish children with hypercholesterolemia. Hum Genet 96: 147-150.

33. Ekstrom U, Abrahamson M, Sveger T, Sun XM, Soutar AK, et al. (2000) Expression of an LDL receptor allele with two different mutations (E256K and I402T). Mol Pathol 53: 31-36.

34. El Messal M, Ait Chihab K, Chater R, Vallve JC, Bennis F, et al. (2003) Familial hypercholesterolemia in Morocco: first report of mutations in the LDL receptor gene. J Hum Genet 48: 199-203.

35. Etxebarria A, Palacios L, Stef M, Tejedor D, Uribe KB, et al. (2012) Functional characterization of splicing and ligand-binding domain variants in the LDL receptor. Hum Mutat 33: 232-243.

36. Fouchier SW, Defesche JC, Umans-Eckenhausen MW, Kastelein JP (2001) The molecular basis of familial hypercholesterolemia in The Netherlands. Hum Genet 109: 602-615.

37. Fouchier SW, Kastelein JJ, Defesche JC (2005) Update of the molecular basis of familial hypercholesterolemia in The Netherlands. Hum Mutat 26: 550-556.

38. Frikke-Schmidt R, Nordestgaard BG, Schnohr P, Tybjaerg-Hansen A (2004) Single nucleotide polymorphism in the low-density lipoprotein receptor is associated with a threefold risk of stroke. A case-control and prospective study. Eur Heart J 25: 943-951.

39. Garcia-Garcia AB, Ivorra C, Martinez-Hervas S, Blesa S, Fuentes MJ, et al. (2011) Reduced penetrance of autosomal dominant hypercholesterolemia in a high percentage of families: importance of genetic testing in the entire family. Atherosclerosis 218: 423-430.

40. Garcia-Garcia AB, Real JT, Puig O, Cebolla E, Marin-Garcia P, et al. (2001) Molecular genetics of familial hypercholesterolemia in Spain: Ten novel LDLR mutations and population analysis. Hum Mutat 18: 458-459.

41. Geisel J, Gielen J, Oette K, Herrmann W, Wielckens K (1998) Mutation analysis of exon 3 of the LDL receptor gene in patients with severe hypercholesterolemia. Clin Chem Lab Med 36: 279-282.

42. Giesel J, Holzem G, Oette K (1995) Screening for mutations in exon 4 of the LDL receptor gene in a German population with severe hypercholesterolemia. Hum Genet 96: 301-304.

43. Gonzalez-Garay ML, McGuire AL, Pereira S, Caskey CT (2013) Personalized genomic disease risk of volunteers. Proc Natl Acad Sci U S A 110: 16957-16962.

44. Górski B, Kubalska J, Naruszewicz M, Lubiński J (1998) LDL-R and Apo-B-100 gene mutations in Polish familial hypercholesterolemias. Hum Genet 102: 562-565.

45. Graham CA, McIlhatton BP, Kirk CW, Beattie ED, Lyttle K, et al. (2005) Genetic screening protocol for familial hypercholesterolemia which includes splicing defects gives an improved mutation detection rate. Atherosclerosis 182: 331-340.

46. Graham CA, Wright WT, McIlhatton BP, Young IS, Nicholls DP (2006) The LDLR variant T705I does not cause the typical phenotype of familial hypercholesterolaemia. Atherosclerosis 188: 218-219.

47. Guardamagna O, Restagno G, Rolfo E, Pederiva C, Martini S, et al. (2009) The type of LDLR gene mutation predicts cardiovascular risk in children with familial hypercholesterolemia. J Pediatr 155: 199-204.e192.

48. Heath KE, Gudnason V, Humphries SE, Seed M (1999) The type of mutation in the low density lipoprotein receptor gene influences the cholesterol-lowering response of the HMG-CoA reductase inhibitor simvastatin in patients with heterozygous familial hypercholesterolaemia. Atherosclerosis 143: 41-54.

49. Hobbs CA, Cleves MA, Keith C, Ghaffar S, James SJ (2005) NKX2.5 and congenital heart defects: A population-based study. Am J Med Genet A 134a: 223-225.

50. Hobbs HH, Brown MS, Goldstein JL (1992) Molecular genetics of the LDL receptor gene in familial hypercholesterolemia. Hum Mutat 1: 445-466.

51. Hobbs HH, Leitersdorf E, Leffert CC, Cryer DR, Brown MS, et al. (1989) Evidence for a dominant gene that suppresses hypercholesterolemia in a family with defective low density lipoprotein receptors. J Clin Invest 84: 656-664.

52. Hobbs HH, Russell DW, Brown MS, Goldstein JL (1990) The LDL receptor locus in familial hypercholesterolemia: mutational analysis of a membrane protein. Annu Rev Genet 24: 133-170.

53. Huijgen R, Kindt I, Defesche JC, Kastelein JJ (2012) Cardiovascular risk in relation to functionality of sequence variants in the gene coding for the low-density lipoprotein receptor: a study among 29,365 individuals tested for 64 specific low-density lipoprotein-receptor sequence variants. Eur Heart J 33: 2325-2330.

54. Huijgen R, Kindt I, Fouchier SW, Defesche JC, Hutten BA, et al. (2010) Functionality of sequence variants in the genes coding for the low-density lipoprotein receptor and apolipoprotein B in individuals with inherited hypercholesterolemia. Hum Mutat 31: 752-760.

55. Humphries SE, Cranston T, Allen M, Middleton-Price H, Fernandez MC, et al. (2006) Mutational analysis in UK patients with a clinical diagnosis of familial hypercholesterolaemia: relationship with plasma lipid traits, heart disease risk and utility in relative tracing. J Mol Med (Berl) 84: 203-214.

56. Humphries SE, Norbury G, Leigh S, Hadfield SG, Nair D (2008) What is the clinical utility of DNA testing in patients with familial hypercholesterolaemia? Curr Opin Lipidol 19: 362-368.

57. Jensen RL, Gillespie D, House P, Layfield L, Shelton C (2004) Endolymphatic sac tumors in patients with and without von Hippel-Lindau disease: the role of genetic mutation, von Hippel-Lindau protein, and hypoxia inducible factor-1alpha expression. J Neurosurg 100: 488-497.

58. Khoo KL, van Acker P, Defesche JC, Tan H, van de Kerkhof L, et al. (2000) Low-density lipoprotein receptor gene mutations in a Southeast Asian population with familial hypercholesterolemia. Clin Genet 58: 98-105.

59. Kim JH, Choi HK, Lee H, Park HY, Kim JH, et al. (2004) Novel and recurrent mutations of the LDL receptor gene in Korean patients with familial hypercholesterolemia. Mol Cells 18: 63-70.

60. King-Underwood L, Gudnason V, Humphries S, Seed M, Patel D, et al. (1991) Identification of the 664 proline to leucine mutation in the low density lipoprotein receptor in four unrelated patients with familial hypercholesterolaemia in the UK. Clin Genet 40: 17-28.

61. Knight BL, Gavigan SJ, Soutar AK, Patel DD (1989) Defective processing and binding of low-density lipoprotein receptors in fibroblasts from a familial hypercholesterolaemic subject. Eur J Biochem 179: 693-698.

62. Koivisto UM, Viikari JS, Kontula K (1995) Molecular characterization of minor gene rearrangements in Finnish patients with heterozygous familial hypercholesterolemia: identification of two common missense mutations (Gly823-->Asp and Leu380-->His) and eight rare mutations of the LDL receptor gene. Am J Hum Genet 57: 789-797.

63. Kolansky DM, Cuchel M, Clark BJ, Paridon S, McCrindle BW, et al. (2008) Longitudinal evaluation and assessment of cardiovascular disease in patients with homozygous familial hypercholesterolemia. Am J Cardiol 102: 1438-1443.

64. Kotze MJ, Langenhoven E, Warnich L, du Plessis L, Marx MP, et al. (1989) The identification of two low-density lipoprotein receptor gene mutations in South African familial hypercholesterolaemia. S Afr Med J 76: 399-401.

65. Kuhrova V, Francova H, Zapletalova P, Freiberger T, Fajkusova L, et al. (2002) Spectrum of low density lipoprotein receptor mutations in Czech hypercholesterolemic patients. Hum Mutat 19: 80.

66. Kuhrova V, Francova H, Zapletalova P, Freiberger T, Fajkusova L, et al. (2001) Spectrum of low density lipoprotein receptor mutations in Czech hypercholesterolemic patients. Hum Mutat 18: 253.

67. Laurie AD, Scott RS, George PM (2004) Genetic screening of patients with familial hypercholesterolaemia (FH): a New Zealand perspective. Atheroscler Suppl 5: 13-15.

68. Lee WK, Haddad L, Macleod MJ, Dorrance AM, Wilson DJ, et al. (1998) Identification of a common low density lipoprotein receptor mutation (C163Y) in the west of Scotland. J Med Genet 35: 573-578.

69. Leigh SE, Foster AH, Whittall RA, Hubbart CS, Humphries SE (2008) Update and analysis of the University College London low density lipoprotein receptor familial hypercholesterolemia database. Ann Hum Genet 72: 485-498.

70. Leren TP, Tonstad S, Gundersen KE, Bakken KS, Rodningen OK, et al. (1997) Molecular genetics of familial hypercholesterolaemia in Norway. J Intern Med 241: 185-194.

71. Lombardi MP, Redeker EJ, Defesche JC, Kamerling SW, Trip MD, et al. (2000) Molecular genetic testing for familial hypercholesterolemia: spectrum of LDL receptor gene mutations in The Netherlands. Clin Genet 57: 116-116.

72. Lombardi P, Sijbrands EJ, Kamerling S, Leuven JA, Havekes LM (1997) The T705I mutation of the low density lipoprotein receptor gene (FH Paris-9) does not cause familial hypercholesterolemia. Hum Genet 99: 106-107.

73. Lombardi P, Sijbrands EJ, van de Giessen K, Smelt AH, Kastelein JJ, et al. (1995) Mutations in the low density lipoprotein receptor gene of familial hypercholesterolemic patients detected by denaturing gradient gel electrophoresis and direct sequencing. J Lipid Res 36: 860.

74. Loubser O, Marais AD, Kotze MJ, Godenir N, Thiart R, et al. (1999) Founder mutations in the LDL receptor gene contribute significantly to the familial hypercholesterolemia phenotype in the indigenous South African population of mixed ancestry. Clin Genet 55: 340-345.

75. Loux N, Saint-Jore B, Collod G, Dairou F, Benlian P, et al. (1992) Screening for new mutations in the LDL receptor gene in seven French familial hypercholesterolemia families by the single strand conformation polymorphism method. Hum Mutat 1: 325-332.

76. Mak YT, Pang CP, Tomlinson B, Zhang J, Chan YS, et al. (1998) Mutations in the low-density lipoprotein receptor gene in Chinese familial hypercholesterolemia patients. Arterioscler Thromb Vasc Biol 18: 1600-1605.

77. Maruyama T, Miyake Y, Tajima S, Harada-Shiba M, Yamamura T, et al. (1995) Common mutations in the low-density-lipoprotein-receptor gene causing familial hypercholesterolemia in the Japanese population. Arterioscler Thromb Vasc Biol 15: 1713-1718.

78. Maruyama T, Yamashita S, Matsuzawa Y, Bujo H, Takahashi K, et al. (2004) Mutations in Japanese subjects with primary hyperlipidemia--results from the Research Committee of the Ministry of Health and Welfare of Japan since 1996. J Atheroscler Thromb 11: 131-145.

79. Mollaki V, Progias P, Drogari E (2013) Novel LDLR Variants in Patients with Familial Hypercholesterolemia: In Silico Analysis as a Tool to Predict Pathogenic Variants in Children and Their Families. Ann Hum Genet 77: 426-34.

80. Mozas P, Castillo S, Tejedor D, Reyes G, Alonso R, et al. (2004) Molecular characterization of familial hypercholesterolemia in Spain: identification of 39 novel and 77 recurrent mutations in LDLR. Hum Mutat 24: 187.

81. Naoumova RP, Neuwirth C, Pottinger B, Whittal R, Humphries SE, et al. (2004) Genetic diagnosis of familial hypercholesterolaemia: a mutation and a rare non-pathogenic amino acid variant in the same family. Atherosclerosis 174: 67-71.

82. Nauck MS, Koster W, Dorfer K, Eckes J, Scharnagl H, et al. (2001) Identification of recurrent and novel mutations in the LDL receptor gene in German patients with familial hypercholesterolemia. Hum Mutat 18: 165-166.

83. Nissen H, Hansen AB, Guldberg P, Hansen TS, Petersen NE, et al. (1998) Evaluation of a clinically applicable mutation screening technique for genetic diagnosis of familial hypercholesterolemia and familial defective apolipoprotein B. Clin Genet 53: 433-439.

84. Pereira E, Ferreira R, Hermelin B, Thomas G, Bernard C, et al. (1995) Recurrent and novel LDL receptor gene mutations causing heterozygous familial hypercholesterolemia in La Habana. Hum Genet 96: 319-322.

85. Punzalan FE, Sy RG, Santos RS, Cutiongco EM, Gosiengfiao S, et al. (2005) Low density lipoprotein--receptor (LDL-R) gene mutations among Filipinos with familial hypercholesterolemia. J Atheroscler Thromb 12: 276-283.

86. Reshef A, Nissen H, Triger L, Hensen TS, Eliav O, et al. (1996) Molecular genetics of familial hypercholesterolemia in Israel. Hum Genet 98: 581-586.

87. Robles-Osorio L, Huerta-Zepeda A, Ordonez ML, Canizales-Quinteros S, Diaz-Villasenor A, et al. (2006) Genetic heterogeneity of autosomal dominant hypercholesterolemia in Mexico. Arch Med Res 37: 102-108.

88. Romano M, Di Taranto MD, Mirabelli P, D'Agostino MN, Iannuzzi A, et al. (2011) An improved method on stimulated T-lymphocytes to functionally characterize novel and known LDLR mutations. J Lipid Res 52: 2095-2100.

89. Rubinsztein DC, Coetzee GA, Marais AD, Leitersdorf E, Seftel HC, et al. (1992) Identification and properties of the proline664-leucine mutant LDL receptor in South Africans of Indian origin. J Lipid Res 33: 1647-1655.

90. Schmidt H, Kostner GM (2000) Familial hypercholesterolemia in Austria reflects the multi-ethnic origin of our country. Atherosclerosis 148: 431-432.

91. Schuster H, Ostwald P, Keller P, Wolfram G, Keller C (1993) Identification of the serine-156 to leucine mutation in the low-density lipoprotein receptor in a German family with familial hypercholesterolemia. Clin Investig 71: 172-175.

92. Soutar AK, Knight BL, Patel DD (1989) Identification of a point mutation in growth factor repeat C of the low density lipoprotein-receptor gene in a patient with homozygous familial hypercholesterolemia that affects ligand binding and intracellular movement of receptors. Proc Natl Acad Sci U S A 86: 4166-4170.

93. Soutar AK, McCarthy SN, Seed M, Knight BL (1991) Relationship between apolipoprotein(a) phenotype, lipoprotein(a) concentration in plasma, and low density lipoprotein receptor function in a large kindred with familial hypercholesterolemia due to the pro664----leu mutation in the LDL receptor gene. J Clin Invest 88: 483-492.

94. Sozen MM, Whittall R, Oner C, Tokatli A, Kalkanoglu HS, et al. (2005) The molecular basis of familial hypercholesterolaemia in Turkish patients. Atherosclerosis 180: 63-71.

95. Sun XM, Patel DD, Knight BL, Soutar AK (1997) Comparison of the genetic defect with LDL-receptor activity in cultured cells from patients with a clinical diagnosis of heterozygous familial hypercholesterolemia. The Familial Hypercholesterolaemia Regression Study Group. Arterioscler Thromb Vasc Biol 17: 3092-3101.

96. Sun XM, Patel DD, Knight BL, Soutar AK (1998) Influence of genotype at the low density lipoprotein (LDL) receptor gene locus on the clinical phenotype and response to lipid-lowering drug therapy in heterozygous familial hypercholesterolaemia. The Familial Hypercholesterolaemia Regression Study Group. Atherosclerosis 136: 175-185.

97. Tada H, Kawashiri MA, Noguchi T, Mori M, Tsuchida M, et al. (2009. A novel method for determining functional LDL receptor activity in familial hypercholesterolemia: application of the CD3/CD28 assay in lymphocytes. Clin Chim Acta 400: 42-47.

98. Taylor A, Tabrah S, Wang D, Sozen M, Duxbury N, et al. (2007) Multiplex ARMS analysis to detect 13 common mutations in familial hypercholesterolaemia. Clin Genet 71: 561-568.

99. Tejedor MT, Cenarro A, Tejedor D, Stef M, Mateo-Gallego R, et al. (2010) Haplotype analyses, mechanism and evolution of common double mutants in the human LDL receptor gene. Mol Genet Genomics 283: 565-574.

100. Thiart R, Varret M, Lintott CJ, Scott RS, Loubser O, et al. (2000) Mutation analysis in a small cohort of New Zealand patients originating from the United Kingdom demonstrates genetic heterogeneity in familial hypercholesterolemia. Mol Cell Probes 14: 299-304.

101. Traeger-Synodinos J, Mavroidis N, Kanavakis E, Drogari E, Humphries SE, et al. (1998) Analysis of low density lipoprotein receptor gene mutations and microsatellite haplotypes in Greek FH heterozygous children: six independent ancestors account for 60% of probands. Hum Genet 102: 343-347.

102. Varret M, Rabes JP, Thiart R, Kotze MJ, Baron H, et al. (1998) LDLR Database (second edition): new additions to the database and the software, and results of the first molecular analysis. Nucleic Acids Res 26: 248-252.

103. Vieira JR, Whittall RA, Cooper JA, Miller GJ, Humphries SE (2006) The A370T variant (StuI polymorphism) in the LDL receptor gene is not associated with plasma lipid levels or cardiovascular risk in UK men. Ann Hum Genet 70: 697-704.

104. Vilades Medel D, Leta Petracca R, Carreras Costa F, Cardona Olle M, Barros Membrilla A, et al. (2013) Coronary computed tomographic angiographic findings in asymptomatic patients with heterozygous familial hypercholesterolemia and null allele low-density lipoprotein receptor mutations. Am J Cardiol 111: 955-961.

105. Wang J, Huff E, Janecka L, Hegele RA (2001) Low density lipoprotein receptor (LDLR) gene mutations in Canadian subjects with familial hypercholesterolemia, but not of French descent. Hum Mutat 18: 359-359.

106. Webb JC, Sun XM, McCarthy SN, Neuwirth C, Thompson GR, et al. (1996) Characterization of mutations in the low density lipoprotein (LDL)-receptor gene in patients with homozygous familial hypercholesterolemia, and frequency of these mutations in FH patients in the United Kingdom. J Lipid Res 37: 368-381.

107. Webb JC, Sun XM, Patel DD, McCarthy SN, Knight BL, et al. (1992) Characterization of two new point mutations in the low density lipoprotein receptor genes of an English patient with homozygous familial hypercholesterolemia. J Lipid Res 33: 689-698.

108. Weiss N, Binder G, Keller C (2000) Mutations in the low-density-lipoprotein receptor gene in German patients with familial hypercholesterolaemia. J Inherit Metab Dis 23: 778-790.

109. Widhalm K, Dirisamer A, Lindemayr A, Kostner G (2007) Diagnosis of families with familial hypercholesterolaemia and/or Apo B-100 defect by means of DNA analysis of LDL-receptor gene mutations. J Inherit Metab Dis 30: 239-247.

110. Zakharova FM, Damgaard D, Mandelshtam MY, Golubkov VI, Nissen PH, et al. (2005) Familial hypercholesterolemia in St-Petersburg: the known and novel mutations found in the low density lipoprotein receptor gene in Russia. BMC Med Genet 6: 6.

111. Zakharova FM, Golubkov VI, Mandel'shtam M, Lipovetskii BM, Gaitskhoki VS (2001) Identification of novel missense mutation G571E, novel silent mutation H229H, nonsense mutation C74X, and four single nucleotide polymorphisms in the low-density lipoprotein receptor in patients with familial hypercholesterolemia from St. Petersburg. Bioorg Khim 27: 393-396.
