# Supplementary material for: Systematic Cell-Based Phenotyping of Missense Alleles Empowers Rare Variant Association Studies: A Case for LDLR and Myocardial Infarction
Source: PLoS Genet. 2015 Feb 3;11(2):e1004855. doi: 10.1371/journal.pgen.1004855 (PMC4409815; doi:10.1371/journal.pgen.1004855)
Supplement: S7 Table — (DOCX) [file pgen.1004855.s014.docx]

| **Table S7. Comparative phenotypes for all *LDLR* missense variants functionally analyzed in this study*** | | | | | | |
| --- | --- | --- | --- | --- | --- | --- |
|  | | | | | | |
|  | | | | | **"total LDL signal“ (overexpression setting)** | |
| **aa**  **change** | **MI** | **LDL-C** | **bioinformatic**  **prediction** | ***in vitro* characterization** | **mean (oe)** | **SD (oe)** |
| G20R | unclear | unclear | benign | benign | 1.04 | 0.17 |
| G48D | benign | benign | pathogenic | benign | 1.21 | 0.10 |
| T62M | unclear | benign | unclear | benign | 0.90 | 0.22 |
| R81C | pathogenic | NA | pathogenic | unclear | 0.68 | 0.09 |
| E101K | NA** | NA | pathogenic | pathogenic | 0.34 | 0.07 |
| P105L | unclear | benign | benign | benign | 1.09 | 0.10 |
| D118Y | pathogenic | NA | unclear | unclear | 0.69 | 0.11 |
| D131G | pathogenic | pathogenic | pathogenic | pathogenic | 0.28 | 0.01 |
| G137S | benign | benign | pathogenic | benign | 0.90 | 0.11 |
| G137V | pathogenic | pathogenic | pathogenic | pathogenic | 0.27 | 0.06 |
| D168N | NA | NA | pathogenic | pathogenic | 0.33 | 0.12 |
| S177L | pathogenic | pathogenic | pathogenic | pathogenic | 0.21 | 0.04 |
| P181R | unclear | unclear | pathogenic | benign | 0.87 | 0.35 |
| Q182R | pathogenic | benign | benign | benign | 0.78 | 0.05 |
| C197R | pathogenic | benign | pathogenic | pathogenic | 0.16 | 0.05 |
| G219D | NA | NA | benign | benign | 1.30 | 0.16 |
| D221G | pathogenic | pathogenic | pathogenic | pathogenic | 0.23 | 0.02 |
| C222Y | pathogenic | NA | pathogenic | pathogenic | 0.07 | 0.05 |
| R237H | benign | benign | unclear | benign | 1.60 | 0.24 |
| R253W | NA | NA | unclear | benign | 1.14 | 0.14 |
| M264L | pathogenic | pathogenic | benign | benign | 0.91 | 0.21 |
| D266E | NA | NA | pathogenic | unclear | 0.59 | 0.11 |
| G269D | pathogenic | benign | benign | benign | 1.16 | 0.08 |
| C276S | pathogenic | pathogenic | pathogenic | pathogenic | 0.31 | 0.06 |
| E277K | unclear | benign | benign | benign | 0.90 | 0.11 |
| F282L | pathogenic | benign | pathogenic | pathogenic | 0.12 | 0.03 |
| H285Y | benign | NA | benign | benign | 0.83 | 0.16 |
| M298V | benign | benign | benign | benign | 1.15 | 0.25 |
| R303Q | pathogenic | benign | benign | unclear | 0.86 | 0.12 |
| G314R | benign | NA | benign | benign | 1.03 | 0.09 |
| N316S | unclear | benign | pathogenic | pathogenic | <<1 | NA |
| G324S | NA | NA | pathogenic | benign | 1.13 | 0.23 |
| N330H | unclear | NA | benign | benign | 1 | 0.16 |
| G335S | unclear | benign | pathogenic | benign | 0.85 | 0.30 |
| D342N | NA | NA | benign | benign | 1.27 | 0.28 |
| G343S | benign | NA | pathogenic | unclear | 0.63 | 0.11 |
| E353K | pathogenic | benign | benign | unclear | 0.65 | 0.11 |
| V369M | benign | benign | pathogenic | benign | 1.14 | 0.09 |
| Q378P | pathogenic | benign | unclear | unclear | 0.64 | 0.06 |
| A391T | NA | NA | benign | benign | 1.31 | 0.25 |
| A399T | unclear | pathogenic | pathogenic | benign | 1.32 | 0.30 |
| R416W | pathogenic | pathogenic | pathogenic | benign | 1.08 | 0.25 |
| L432V | pathogenic | pathogenic | pathogenic | benign | 0.76 | 0.13 |
| L446V | pathogenic | pathogenic | benign | benign | 0.95 | 0.10 |
| G461C | pathogenic | NA | benign | benign | 0.90 | 0.12 |
| Y465N | pathogenic | pathogenic | benign | benign | 0.87 | 0.12 |
| V468I | pathogenic | benign | benign | benign | 0.77 | 0.16 |
| D472Y | pathogenic | pathogenic | pathogenic | pathogenic | 0.63 | 0.13 |
| D492N | pathogenic | benign | pathogenic | benign | 0.97 | 0.16 |
| K504E | pathogenic | pathogenic | benign | benign | 1.14 | 0.13 |
| G516D | benign | benign | benign | benign | 1.18 | 0.28 |
| V523M | pathogenic | NA | pathogenic | benign | 0.81 | 0.17 |
| V524M | pathogenic | benign | pathogenic | benign | 1.08 | 0.21 |
| P526S | benign | pathogenic | pathogenic | pathogenic | <<1 | NA |
| V527A | benign | NA | benign | benign | 0.90 | 0.13 |
| G529R | pathogenic | benign | pathogenic | benign | 0.80 | 0.12 |
| G549D | pathogenic | pathogenic | pathogenic | pathogenic | 0.06 | 0.04 |
| R574C | pathogenic | pathogenic | pathogenic | benign | 1.12 | 0.08 |
| H583D | pathogenic | pathogenic | pathogenic | pathogenic | 0.17 | 0.05 |
| D589H | pathogenic | benign | pathogenic | benign | 1.08 | 0.12 |
| G592E | unclear | pathogenic | pathogenic | benign | 0.95 | 0.17 |
| R595Q | pathogenic | unclear | pathogenic | benign | 0.85 | 0.20 |
| A606S | pathogenic | benign | benign | benign | 1.16 | 0.09 |
| E626K | pathogenic | benign | benign | unclear | 0.62 | 0.14 |
| D651N | pathogenic | NA | pathogenic | benign | 0.71 | 0.08 |
| M652V | benign | NA | benign | benign | 1.07 | 0.20 |
| P685L | pathogenic | pathogenic | pathogenic | pathogenic | 0.18 | 0.06 |
| G701S | unclear | benign | pathogenic | unclear | 0.69 | 0.15 |
| R706G | benign | benign | benign | benign | 1.20 | 0.22 |
| R709K | pathogenic | benign | unclear | benign | 0.85 | 0.17 |
| T726I | unclear | unclear | benign | benign | 1.51 | 0.22 |
| R744Q | unclear | benign | benign | benign | 1.02 | 0.07 |
| D748N | pathogenic | pathogenic | benign | unclear | 1.15 | 0.19 |
| T761M | pathogenic | benign | pathogenic | benign | 1.03 | 0.20 |
| S786G | benign | NA | benign | benign | 1.15 | 0.30 |
| V800I | benign | benign | benign | benign | 1.06 | 0.22 |
| R814Q | NA | NA | unclear | benign | 0.81 | 0.08 |
| V827I | benign | NA | pathogenic | benign | 1.03 | 0.11 |
| Y828C | NA | NA | pathogenic | unclear | 0.65 | 0.20 |
| V859M | pathogenic | pathogenic | benign | NA | NA | NA |
| ***** *LDLR* missense variants functionally characterized in this study were classified according to  (i) occurrence in MI versus MI-free individuals (“pathogenic”: variant only found among MI cases; “unclear”: variant is present in MI cases as well as MI controls; “benign”: only found among MI-free controls;  (ii) occurrence in individuals with highly elevated plasma LDL-C (>190mg/dl): “pathogenic”: the mean of all individuals with this variant was >190mg/dl; “unclear”: at least one carrier showed LDL-C>190mg/dl; “benign”: LDL-C of all individuals <190mg/dl;  (iii) functional impact as predicted by the overlap of four bioinformatic prediction tools (“Prediction summary”): PolyPhen-2, SIFT, MutationAssessor and MutationTaster (see Methods).  (iv) functional characterisation performed within this study: “pathogenic”: disruptive-missense (FH-like), “benign”: non-disruptive (FH-unlike) (for details of classification: see Methods).  Amino acid numbers refer to LDLR RefSeq transcript NM_000527.4 (ENST00000558518; 860aa), **NA: not available | | | | | | |
