# Supplementary material for: Systematic Cell-Based Phenotyping of Missense Alleles Empowers Rare Variant Association Studies: A Case for LDLR and Myocardial Infarction
Source: PLoS Genet. 2015 Feb 3;11(2):e1004855. doi: 10.1371/journal.pgen.1004855 (PMC4409815; doi:10.1371/journal.pgen.1004855)
Supplement: S8 Table — (DOCX) [file pgen.1004855.s015.docx]

| **Table S8. Common variants used to determine polygenic risk scores for association with plasma LDL-C.** | | | |
| --- | --- | --- | --- |
|  |  |  |  |
| **no.** | **chr. position** | **gene** | **variant (rsID)** |
| 1 | 1: 25775733 | LDLRAP1 | rs12027135 |
| 2 | 1: 55504650 | PCSK9 | rs2479409 |
| 3 | 1: 63025942 | ANGPTL3 | rs2131925 |
| 4 | 2: 21263900 | APOB | rs1367117 |
| 5 | 2: 44072576 | ABCG5/8 | rs4299376 |
| 6 | 6: 16127407 | MYLIP | rs3757354 |
| 7 | 6: 26093141 | HFE | rs1800562 |
| 8 | 6: 32412435 | HLA | rs3177928 |
| 9 | 6: 160578860 | LPA | rs1564348 |
| 10 | 7: 21607352 | DNAH11 | rs12670798 |
| 11 | 7: 44579180 | NPC1L1 | rs2072183 |
| 12 | 10: 113933886 | GPAM | rs2255141 |
| 13 | 11: 61569830 | FADS1–2–3 | rs174546 |
| 14 | 12: 112072424 | BRAP | rs11065987 |
| 15 | 12: 121416650 | HNF1A | rs1169288 |
| 16 | 14: 24883887 | NYNRIN | rs8017377 |
| 17 | 16: 56993324 | CETP | rs3764261 |
| 18 | 16: 72108093 | HPR | rs2000999 |
| 19 | 19: 11202306 | LDLR | rs6511720 |
| 20 | 19: 19407718 | CILP2 | rs10401969 |
