# Supplementary material for: Systematic Cell-Based Phenotyping of Missense Alleles Empowers Rare Variant Association Studies: A Case for LDLR and Myocardial Infarction
Source: PLoS Genet. 2015 Feb 3;11(2):e1004855. doi: 10.1371/journal.pgen.1004855 (PMC4409815; doi:10.1371/journal.pgen.1004855)
Supplement: S9 Table — (DOCX) [file pgen.1004855.s016.docx]

| **Table S9. Rare and low-frequency coding variants identified in 12 Mendelian lipid disease genes among 23 LDLR variant carriers with unexpected high or low plasma LDL-C.** | | | | | | | | |
| --- | --- | --- | --- | --- | --- | --- | --- | --- |
|  | | | | | | | | |
|  | **gene** | **chr. position** | **ref** | **alt** | **variant** | **MAF*** | **no. indiv.** | **plasma LDL-C [mg/dl]** |
| RARE VARIANTS (MAF<0.01) | ABCG5 | 2:44059195 | G | C | A17G | 0,009597523 | 1 | 131,8 |
|  | ABCG5 | 2:44065739 | C | G | G27A | 0,00927357 | 1 | 223 |
|  | ABCG8 | 2:44079930 | C | T | A296V | 0,000309502 | 1 | 324 |
|  | APOB | 2:21232455 | A | T | S2429T | 0,003102699 | 1 | 180,8 |
|  | APOB | 2:21230858 | T | C | N2961S | 0,000463822 | 1 | 129 |
|  | APOE | 19:45411987 | G | A | G145D** | 0,003274088 | 1 | 206,2 |
|  | NPC1L1 | 7:44574063 | A | G | F717L | 0,00015456 | 1 | 220 |
|  | NPC1L1 | 7:44555699 | A | T | I1160N | 0,004018547 | 1 | 131,8 |
| LOW FREQ. VARIANTS (MAF<0.05) | APOA5 | 11:116661488 | C | T | V153M | 0,031993818 | 1 | 131,8 |
|  | APOB | 2:21249716 | C | T | V730I | 0,031530139 | 3 | 72; 295.8; 324 |
|  | APOB | 2:21233972 | T | C | H1923R | 0,02812983 | 2 | 139.6; 220 |
|  | APOB | 2:21225485 | C | G | R4270T | 0,014683153 | 1 | 206,2 |
|  | APOB | 2:21224853 | C | T | A4481T | 0,046213292 | 3 | 129; 309.4; 214.4 |
|  | LDLRAP1 | 1:25890247 | C | T | R238W*** | 0,048840804 | 2 | 72; 197 |
|  | MTTP | 4:100504575 | G | C | E108D | 0,031993818 | 2 | 214.4; 198.6 |
|  | MTTP | 4:100510903 | A | G | N166S | 0,031993818 | 2 | 214.4; 198.6 |
|  | MTTP | 4:100512392 | G | A | V168I | 0,011128284 | 1 | 180,8 |
|  | MTTP | 4:100512919 | C | G | Q244E | 0,025193199 | 3 | 110.8; 278.6; 223 |
|  | MTTP | 4:100521805 | A | C | D384A | 0,044358578 | 1 | 197 |
|  | MTTP | 4:100532602 | G | A | G661S | 0,049768161 | 2 | 180.8; 295.8 |
|  | PCSK9 | 1:55529187 | G | A | G670E | 0,033848532 | 1 | 139,6 |
| *MAF, minor allele frequency; **variant previously identified in patients;  ***variant previously described as non-disease related polymorphism | | | | | | | | |
