# Supplementary material for: Systematic Cell-Based Phenotyping of Missense Alleles Empowers Rare Variant Association Studies: A Case for LDLR and Myocardial Infarction
Source: PLoS Genet. 2015 Feb 3;11(2):e1004855. doi: 10.1371/journal.pgen.1004855 (PMC4409815; doi:10.1371/journal.pgen.1004855)
Supplement: S10 Table — (DOCX) [file pgen.1004855.s017.docx]

| **Table S10. Distribution of MI risk factors between ATVB MI cases and controls (means).** | | |
| --- | --- | --- |
|  |  |  |
|  | **MI controls** | **MI cases** |
| **Trait** | **(n=1,519)** | **(n=1,716)** |
| LDL [mg/dl] | 125,5 | 147,8 |
| HDL [mg/dl] | 48,9 | 41,8 |
| TG [mg/dl] | 121,1 | 178,3 |
| TC [mg/dl] | 201,4 | 221,4 |
| T2D [% individu  als] | 0,90% | 7,80% |
| BMI | 25 | 26,8 |
| weight [kg] | 76,1 | 79,6 |
| height [cm] | 174,3 | 172 |
| SBP [mmHg] | 123,7 | 132,2 |
| DBP [mmHg] | 79,7 | 83,4 |
| current smoker [%] | 31,10% | 45,50% |
| former smoker [%] | 17,30% | 42,30% |
| age (years) | 39,6 | 39,6 |
