# Supplementary material for: Systematic Cell-Based Phenotyping of Missense Alleles Empowers Rare Variant Association Studies: A Case for LDLR and Myocardial Infarction
Source: PLoS Genet. 2015 Feb 3;11(2):e1004855. doi: 10.1371/journal.pgen.1004855 (PMC4409815; doi:10.1371/journal.pgen.1004855)
Supplement: S11 Table — (DOCX) [file pgen.1004855.s018.docx]

| **Table S11. Primer sequences for site-directed mutagenesis of LDLR'-GFP*** | | | | | |
| --- | --- | --- | --- | --- | --- |
|  | | | | | |
|  |  |  |  | **oligonucleotides used for site-directed mutagenesis of LDLR'-GFP** | |
| **pos** | **ref** | **alt** | **aa chg** | **forward** | **reverse** |
| 19:11200282 | G | A | G20R | tcgccgcggcgaggactgcagtg | cactgcagtcctcgccgcggcga |
| 19:11210974 | G | A | G48D | tgggtctgcgatgacagcgctgagtgc | gcactcagcgctgtcatcgcagaccca |
| 19:11211016 | C | T | T62M | gatgagtcccaggagatgtgcttgtctgtcacc | ggtgacagacaagcacatctcctgggactcatc |
| 19:11213390 | C | T | R81C | ggggccgtgtcaactgctgcattcctcag | ctgaggaatgcagcagttgacacggcccc |
| 19:11213450 | G | A | E101K | caacggctcagacaagcaaggctgtccc | gggacagccttgcttgtctgagccgttgt |
| 19:11215896 | C | T | P105L | gagcaaggctgtctccccaagacgtgc | gcacgtcttggggagacagccttgctc |
| 19:11215934 | G | T | D118Y | cgagtttcgctgccactatgggaagtgcatctc | gagatgcacttcccatagtggcagcgaaactcg |
| 19:11215974 | A | G | D131G | cgtctgtgactcaggccgggactgcttgg | ccaagcagtcccggcctgagtcacagacg |
| 19:11215991 | G | A | G137S | ggactgcttggacagctcagacgaggc | gcctcgtctgagctgtccaagcagtcc |
| 19:11215992 | G | T | G137V | gactgcttggacgtctcagacgaggcc | ggcctcgtctgagacgtccaagcagtc |
| 19:11216064 | G | A | D168N | ctgtgggcctgcaacaacgaccccg | cggggtcgttgttgcaggcccacag |
| 19:11216112 | C | T | S177L | gactgcgaagatggcttggatgagtggccg | cggccactcatccaagccatcttcgcagtc |
| 19:11216124 | C | G | P181R | cggatgagtggcggcagcgctgtag | ctacagcgctgccgccactcatccg |
| 19:11216127 | A | G | Q182R | atgagtggccgcggcgctgtagggg | cccctacagcgccgcggccactcat |
| 19:11216171 | T | C | C197R | gacagtagcccccgctcggccttcg | cgaaggccgagcgggggctactgtc |
| 19:11216238 | G | A | G219D | cgctgtgatggtgaccccgactgcaag | cttgcagtcggggtcaccatcacagcg |
| 19:11216244 | A | G | D221G | tgatggtggccccggctgcaaggacaaatc | gatttgtccttgcagccggggccaccatca |
| 19:11216247 | G | A | C222Y | gatggtggccccgactacaaggacaaatctgac | gtcagatttgtccttgtagtcggggccaccatc |
| 19:11217256-7 | GG | AC | R237H | agccggcagtgtgaccacgaatatgactgcaagg | ccttgcagtcatattcgtggtcacactgccggct |
| 19:11217303 | C | T | R253W | catccatggcagctggcagtgtgaccg | cggtcacactgccagctgccatggatg |
| 19:11217336 | A | C | M264L | aatatgactgcaaggacctgagcgatgaagttggc | gccaacttcatcgctcaggtccttgcagtcatatt |
| 19:11217344 | T | A | D266E | gcaaggacatgagcgaagaagttggctgcgtta | taacgcagccaacttcttcgctcatgtccttgc |
| 19:11217352 | G | A | G269D | catgagcgatgaagttgactgcgttaatgtgacac | gtgtcacattaacgcagtcaacttcatcgctcatg |
| 19:11218077 | G | C | C276S | tgcgttaatgtgacactctccgagggaccc | gggtccctcggagagtgtcacattaacgca |
| 19:11218079 | G | A | E277K | ttaatgtgacactctgcaagggacccaacaagttc | gaacttgttgggtcccttgcagagtgtcacattaa |
| 19:11218096 | C | A | F282L | tctgcgagggacccaacaagttaaagtgtcacagc | gctgtgacactttaacttgttgggtccctcgcaga |
| 19:11218103 | C | T | H285Y | ggacccaacaagttcaagtgttacagcggcgaa | ttcgccgctgtaacacttgaacttgttgggtcc |
| 19:11218142 | A | G | M298V | ggacaaagtctgcaacgtggctagagactgccg | cggcagtctctagccacgttgcagactttgtcc |
| 19:11218158 | G | A | R303Q | ggctagagactgccaggactggtcagatg | catctgaccagtcctggcagtctctagcc |
| 19:11218190 | G | A | G314R | aacccatcaaagagtgcaggaccaacgaatgcttg | caagcattcgttggtcctgcactctttgatgggtt |
| 19:11221334 | A | G | N316S | caaagagtgcgggaccagcgaatgcttggac | gtccaagcattcgctggtcccgcactctttg |
| 19:11221357 | G | A | G324S | tgcctgtgccccaacggcttccagc | gctggaagccgttggggcacaggca |
| 19:11221375 | A | C | N330H | ctgttcccacgtctgccatgaccttaagatcgg | ccgatcttaaggtcatggcagacgtgggaacag |
| 19:11221390 | G | A | G335S | gcaatgaccttaagatcagctacgagtgcctgtgc | gcacaggcactcgtagctgatcttaaggtcattgc |
| 19:11221411 | G | A | D342N | tggacaacaacggcagctgttcccacgtc | gacgtgggaacagctgccgttgttgtcca |
| 19:11221414 | G | A | G343S | ctgtgccccgacagcttccagctgg | ccagctggaagctgtcggggcacag |
| 19:11221444 | G | A | E353K | ggcccagcgaagatgcaaagatatcgatgagtg | cactcatcgatatctttgcatcttcgctgggcc |
| 19:11222234 | G | A | V369M | cagccagctctgcatgaacctggaggg | ccctccaggttcatgcagagctggctg |
| 19:11222262 | A | C | Q378P | gtggctacaagtgcccgtgtgaggaaggctt | aagccttcctcacacgggcacttgtagccac |
| 19:11222300 | G | A | A391T | cccccacacgaagacctgcaaggctgt | acagccttgcaggtcttcgtgtggggg |
| 19:11223962 | G | A | A399T | gctgtgggctccatcacctacctcttcttca | tgaagaagaggtaggtgatggagcccacagc |
| 19:11224013 | C | T | R416W | agatgacgctggactggagcgagtacacc | ggtgtactcgctccagtccagcgtcatct |
| 19:11224061 | C | G | L432V | gaggaacgtggtcgctgtggacacgg | ccgtgtccacagcgaccacgttcctc |
| 19:11224103 | C | G | L446V | gaatctactggtctgacgtgtcccagagaatgatc | gatcattctctgggacacgtcagaccagtagattc |
| 19:11224233 | G | T | G461C | cttgacagagcccactgcgtctcttcctatg | cataggaagagacgcagtgggctctgtcaag |
| 19:11224245 | T | A | Y465N | cacggcgtctcttccaatgacaccgtcatca | tgatgacggtgtcattggaagagacgccgtg |
| 19:11224254 | G | A | V468I | tctcttcctatgacaccatcatcagcagagacatc | gatgtctctgctgatgatggtgtcataggaagaga |
| 19:11224266 | G | T | D472Y | caccgtcatcagcagatacatccaggccc | gggcctggatgtatctgctgatgacggtg |
| 19:11224326 | G | A | D492N | caacatctactggaccaactctgtcctgggcac | gtgcccaggacagagttggtccagtagatgttg |
| 19:11224362 | A | G | K504E | ctgttgcggataccgagggcgtgaagagg | cctcttcacgccctcggtatccgcaacag |
| 19:11224399 | G | A | G516D | tattcagggagaacgactccaagccaagggc | gcccttggcttggagtcgttctccctgaata |
| 19:11224419 | G | A | V523M | aagccaagggccatcatggtggatcctgttc | gaacaggatccaccatgatggcccttggctt |
| 19:11224422 | G | A | V524M | ccaagggccatcgtgatggatcctgttcatg | catgaacaggatccatcacgatggcccttgg |
| 19:11224428 | C | T | P526S | ggccatcgtggtggattctgttcatggcttca | tgaagccatgaacagaatccaccacgatggcc |
| 19:11224432 | T | C | V527A | catcgtggtggatcctgctcatggcttcatgtact | agtacatgaagccatgagcaggatccaccacgatg |
| 19:11224437 | G | C | G529R | gtggtggatcctgttcatcgcttcatgtactgga | tccagtacatgaagcgatgaacaggatccaccac |
| 19:11226829 | G | A | G549D | aaagggggcctgaatgatgtggacatctactcg | cgagtagatgtccacatcattcaggcccccttt |
| 19:11227549 | C | T | R574C | gatctcctcagtggctgcctctactgggttg | caacccagtagaggcagccactgaggagatc |
| 19:11227576 | C | G | H583D | ctgggttgactccaaacttgactccatctcaagc | gcttgagatggagtcaagtttggagtcaacccag |
| 19:11227594 | G | C | D589H | ccatctcaagcatccatgtcaacgggggc | gcccccgttgacatggatgcttgagatgg |
| 19:11227604 | G | A | G592E | gcatcgatgtcaacgagggcaaccggaagac | gtcttccggttgccctcgttgacatcgatgc |
| 19:11227613 | G | A | R595Q | caacgggggcaaccagaagaccatcttgg | ccaagatggtcttctggttgcccccgttg |
| 19:11227645 | G | T | A606S | gatgaaaagaggctgtcccaccccttctcct | aggagaaggggtgggacagcctcttttcatc |
| 19:11230798 | G | A | E626K | tttggacagatatcatcaacaaagccattttcagtgccaac | gttggcactgaaaatggctttgttgatgatatctgtccaaa |
| 19:11230873 | G | A | D651N | acctactgtccccagagaatatggttctcttccac | gtggaagagaaccatattctctggggacagtaggt |
| 19:11230876 | A | G | M652V | cctactgtccccagaggatgtggttctcttcca | tggaagagaaccacatcctctggggacagtagg |
| 19:11231112 | C | T | P685L | gcctccctgccctgcagatcaaccc- | gggttgatctgcagggcagggaggc |
| 19:11231159 | G | A | G701S | gcctgcccggacagcatgctgctgg | ccagcagcatgctgtccgggcaggc |
| 19:11231174 | A | G | R706G | catgctgctggccggggacatgaggag | ctcctcatgtccccggccagcagcatg |
| 19:11231184 | G | A | R709K | ggccagggacatgaagagctgcctcacag | ctgtgaggcagctcttcatgtccctggcc |
| 19:11233886 | C | T | T726I | acccaggagacatccatcgtcaggctaaagg | cctttagcctgacgatggatgtctcctgggt |
| 19:11233940 | G | A | R744Q | gcacacaaccacccaacctgttcccgaca | tgtcgggaacaggttgggtggttgtgtgc |
| 19:11233951 | G | A | D748N | cgacctgttcccaacacctcccggc | gccgggaggtgttgggaacaggtcg |
| 19:11233991 | C | T | T761M | cccctgggctcaccatggtggagatagtg | cactatctccaccatggtgagcccagggg |
| 19:11238728 | A | G | S786G | ggaaatgagaagaagcccagtggcgtgagggct | agccctcacgccactgggcttcttctcatttcc |
| 19:11240197 | G | A | V800I | cccatcgtgctcctcatcttcctttgcctgg | ccaggcaaaggaagatgaggagcacgatggg |
| 19:11240240 | G | A | R814Q | cttctatggaagaactggcagcttaagaacatcaacagc | gctgttgatgttcttaagctgccagttcttccatagaag |
| 19:11240278 | G | A | V827I | atcaactttgacaaccccatctatcagaagaccacag | ctgtggtcttctgatagatggggttgtcaaagttgat |
| 19:11240282 | A | G | Y828C | aactttgacaaccccgtctgtcagaagaccacag | ctgtggtcttctgacagacggggttgtcaaagtt |
| 19:11241984 | G | A | V859M | NA | NA |
| * siRNA-oligonucleotides were fluid phase transfected using Oligofectamine to knock-down endogenous LDLR-protein levels  (for details see Methods). Amino acid numbers refer to LDLR RefSeq transcript NM_000527.4 (ENST00000558518; 860aa). | | | | | |
